# Bone-targeted erythrocyte-cancer hybrid membrane-camouflaged nanoparticles for enhancing photothermal and hypoxia-activated chemotherapy of bone invasion by OSCC

**DOI:** 10.1186/s12951-021-01088-9

**Published:** 2021-10-26

**Authors:** Hongying Chen, Jiang Deng, Xintong Yao, Yungang He, Hanyue Li, Zhixiang Jian, Yi Tang, Xiaoqing Zhang, Jingqing Zhang, Hongwei Dai

**Affiliations:** 1 College of Stomatology, Chongqing Medical University, Chongqing, 401147 China; 2 Chongqing Key Laboratory of Oral Diseases and Biomedical Sciences, Chongqing, 401147 China; 3 Chongqing Municipal Key Laboratory of Oral Biomedical Engineering of Higher Education, Chongqing, 401147 China; 4 Department of Pharmacology, School of Pharmacy, Chongqing Medical University, Chongqing, 400016 China; 5 Key Laboratory of Biochemistry and Molecular Pharmacology of Chongqing, Chongqing Medical University, Chongqing, 400016 China; 6 Chongqing Research Center for Pharmaceutical Engineering, Chongqing Medical University, Chongqing, 400016 China

**Keywords:** Hybrid membrane, Homing-targeting, Bone targeting, Biomimetic nanoparticle, Drug delivery

## Abstract

**Background:**

Jaw bones are the most common organs to be invaded by oral malignancies, such as oral squamous cell carcinoma (OSCC), because of their special anatomical relationship. Various serious complications, such as pathological fractures and bone pain can significantly decrease the quality of life or even survival outcomes for a patient. Although chemotherapy is a promising strategy for bone invasion treatment, its clinical applications are limited by the lack of tumor-specific targeting and poor permeability in bone tissue. Therefore, it is necessary to develop a smart bone and cancer dual targeting drug delivery platform.

**Results:**

We designed a dual targeting nano-biomimetic drug delivery vehicle Asp8[H40-TPZ/IR780@(RBC-H)] that has excellent bone and cancer targeting as well as immune escape abilities to treat malignancies in jaw bones. These nanoparticles were camouflaged with a head and neck squamous cell carcinoma WSU-HN6 cell (H) and red blood cell (RBC) hybrid membrane, which were modified by an oligopeptide of eight aspartate acid (Asp8). The spherical morphology and typical core-shell structure of biomimetic nanoparticles were observed by transmission electron microscopy. These nanoparticles exhibited the same surface proteins as those of WSU-HN6 and RBC. Flow cytometry and confocal microscopy showed a greater uptake of the biomimetic nanoparticles when compared to bare H40-PEG nanoparticles. Biodistribution of the nanoparticles in vivo revealed that they were mainly localized in the area of bone invasion by WSU-HN6 cells. Moreover, the Asp8[H40-TPZ/IR780@(RBC-H)] nanoparticles exhibited effective cancer growth inhibition properties when compared to other TPZ or IR780 formulations.

**Conclusions:**

Asp8[H40-TPZ/IR780@(RBC-H)] has bone targeting, tumor-homing and immune escape abilities, therefore, it is an efficient multi-targeting drug delivery platform for achieving precise anti-cancer therapy during bone invasion.

**Graphical Abstract:**

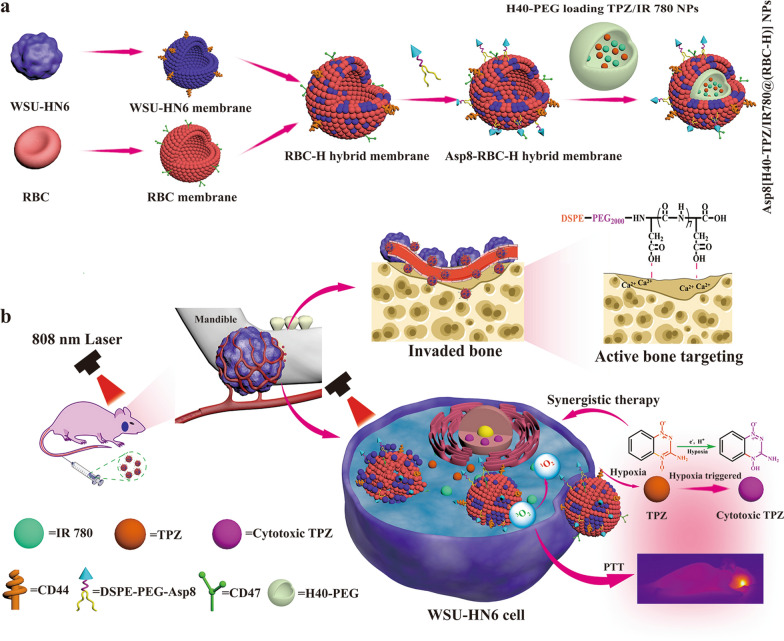

**Supplementary Information:**

The online version contains supplementary material available at 10.1186/s12951-021-01088-9.

## Background

Due to the specific anatomical relationship between soft tissues and the jaw bones in the oral cavity, bone invasion has become one of the most frequent complication of oral squamous cell carcinoma (OSCC) [1–3]. Moreover, bone invasion can lead to OSCC relapse, which can significantly impact on patient prognosis and their quality of life [3, 4]. Serious osteolytic lesions, which occur between the bone microenvironment and OSCC, indicate bone invasion. Pathological fracture, hypocalcemia and serious pain are associated with advanced OSCC [5]. Although surgery and chemotherapy are treatment options for bone invasion by OSCC, they do not exhibit satisfactory outcomes [6]. To treat OSCC bone invasion, cancer cell growth should be inhibited in the bone lesion area to suppress bone resorption. A combined approach that can target both the damaging microenvironment of bone and the OSCC cells, will provide superior therapeutic effects than the single therapeutic options and improve the prognosis of advance OSCC [7].

Advances in biomedical nanoparticles make targeted delivery, sustained and controlled release of therapeutic compounds possible within tumor tissues [8]. In addition, nanoparticles as carriers for therapeutic agents are associated with several advantages, including increased solubility, extended circulation time, and avoiding degradation of the agent [9–11]. Therefore, nanoparticles have been widely evaluated as drug delivery vehicles in carcinoma diagnosis and treatment [12]. Targeted drug delivery systems involving nanotechnology fabrication have exhibited a greater potential [13]. Although nanoparticles have been used to design various types of vehicles for improving therapeutic efficacies, their efficiencies in clinical applications have not been clearly established.

Low targeting capacities and inefficient circulation time inhibit the application of nano-based drug delivery vehicles [14]. To solve these challenges, an approach that involves ligand-related surface decoration by a chemical reaction has been used to escape immune recognition and clearance [15]. However, this approach did not show satisfactory outcomes. In 2011, Zhang and co-workers reported a new category of drug vehicle, which was prepared by extruding the erythrocyte membrane and a polymeric nanoparticle (PLGA) [16]. The cell membrane-camouflaged approach endows PLGA with biointerface properties, such as the CD47 membrane protein, which is obtained from the cellular membrane. Unique biointerfacing can make PLGA to escape recognition and clearance by the immune system. In addition, biomimetic nanoparticles can also enhance specific targeting capabilities to carcinoma cells by camouflaging the cancer cell membrane [17]. Apart from cancer cells and erythrocytes, leukocytes, stem cells as well as platelets are also used to construct biomimetic nanoparticles to realize synthetic nanoparticle functionalization [18]. However, individual cell membrane-camouflaged biomimetic nanoparticles only exhibit single properties, such as immune evasion or homologous targeting abilities. To realize multifunctional delivery vehicles, studies have focused on hybrid cell membrane-coated nanoparticles [19]. In 2017, Zhang et al. reported a novel approach for constructing hybrid cell membrane-camouflaged nanoparticles by fusing RBCs and platelets membrane and coating both cell membrane segments on PLGA nanoparticles. The formed biomimetic nanoparticles were shown multiple tasks in complex biological microenvironments [20]. Sun et al. reported a strategy for fusing cancer stem cell membranes and plate membranes on iron oxide magnetic nanoparticle surfaces to enhance magnetic nanoparticle functions [21]. The resulting biomimetic nanoparticle exhibited superior homologous cancer cell targeting and immune evasion capacities.

The hybrid membrane coating strategy has been important in improving applications of synthetic nanoparticles [22]. However, due to lacking bone targeting abilities, biomimetic drug delivery vehicles often fail to accumulate in sites of bone lesion, leading to the release of inadequate dose of therapeutic drugs for cancer. Due to non-specific biodistribution of therapeutic drugs, patients are subjected to serious adverse effects [23]. Therefore, it is necessary to improve target accumulation abilities of various nanoparticles. Cheng and co-workers summarized approaches in the design of the bone-targeted nanoparticles in detail. And kinds of bone-targeted ligands were described by diagrams. These nanoparticles could be precisely located in bone resorption sites due to the navigation of targeting ligands [24]. However, among the targeting ligands, the use of bisphosphonates (BPs) is limited due to a serious side-effect of bisphosphonate-related osteonecrosis of the jaw (BRONJ) [25]. Except for the BPs, it is reported that osteocalcin and osteopontin also have a high affinity for binding to bone tissues owing to be comprised by abundant aspartic acid (Asp) [24, 26]. Therefore, Asp-rich peptides, such as Asp6, Asp7 and Asp8, has been used as bone-targeting ligands to decorate drug delivery vehicles [27]. Cheng et al. developed an octapeptide (Asp8) decorated dendritic platinum-copper alloy drug delivery system (Asp-DPCN). The NPs not only showed satisfying binding affinities to hydroxyapatite (HA), but also had excellent photothermal conversion capability in both in vitro and in vivo experiments [28]. Moreover, Jiang and co-workers designed a biomimetic targeting drug delivery platform (PDA-SN38@SCM) which was composed of the inner core of loaded anticancer drug (SN38) polydopamine nanoparticle and outer shell of umbilical-cord mesenchymal stem cell membrane. And these biomimetic NPs exhibited excellent photothermal effect and bone tumor targeting property [29]. Compared to unmodified delivery vehicles, the amount of delivery vehicles that bound hydroxyapatite was increased by about 14-fold after Asp8 modification [30, 31]. Therefore, bone-targeted drug delivery vehicles can deliver the anticancer drugs to bone lesion sites [27, 32]. However, to the best of our knowledge, it has not been determined whether synthetic nanoparticle functionality could be enhanced by fusing both erythrocyte and OSCC cell membranes and coupling them with a bone targeting ligand.

**Scheme 1:**
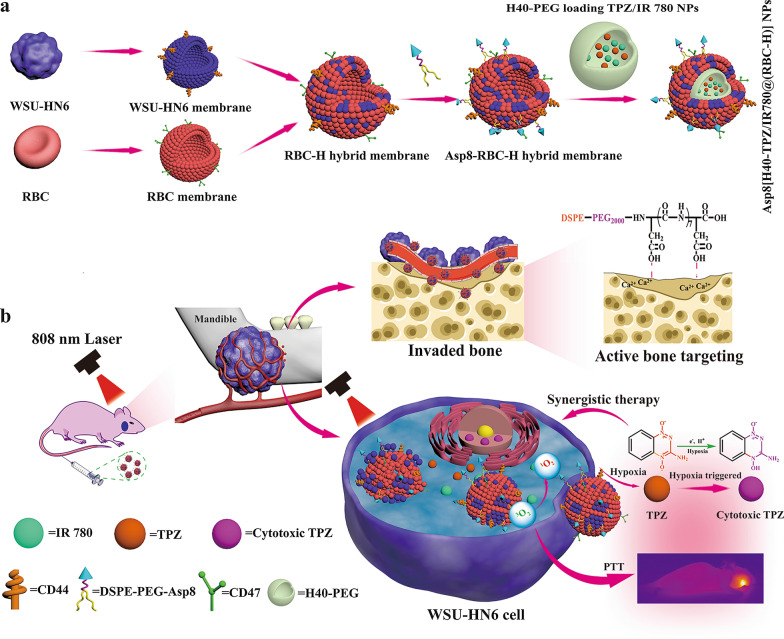
Schematic illustration of Asp8-modified RBC-H hybrid membrane-coated H40-TPZ/IR780 NPs for enhancing photothermal and chemotherapy of bone invasion by OSCC. **a** Preparation of the Asp8[H40-TPZ/IR780@(RBC-H)] nanoparticle. **b** Targeting delivery TPZ and IR780 to bone invasion site by the resulting nanoparticle for enhancing photothermal and hypoxia-activated synergistic chemotherapy effects in vivo

We report a strategy for fusing RBC and WSU-HN6 cell membranes to construct a RBC-H hybrid exterior shell, and successfully camouflage them onto TPZ and IR780-loaded hyperbranched polymer nanoparticles (Asp8[H40-TPZ/IR780@(RBC-H)] NPs) for treatment of bone invasion (Scheme 1). As a targeting mitochondrial photosensitizer, released IR780 not only exhibits highly efficient photothermal conversion property, but also have singlet oxygen (^1^O_2_) generation capacity, upon an 808 nm laser irradiation. Furthermore, IR780 can further exacerbate tumor hypoxia microenvironment during photothermal treatment (PTT) process. Subsequently, as a hypoxia-activated prodrug, TPZ released from biomimetic NPs could be activated to produce toxic oxidizing radicals (benzotriazinyl radical and hydroxyl radical) to exert anticancer effects under the hypoxia microenvironment. In this study, the biomimetic NPs were characterized, and it was revealed that they exhibited characteristic functionality from both erythrocytes and WSU-HN6 cells. Due to the CD47 protein marker of RBC membranes, immune escaping abilities of camouflaged H40-TPZ/IR780 nanoparticles were significantly improved and they were able to escape macrophage phagocytosis [33]. In addition, because of its unique cell membrane proteins, such as CD44, the WSU-HN6 cell membrane coating enhanced OSCC cell homologous targeting ability. Compared to bare H40-PEG NPs, biomimetic NPs exhibited stronger specific self-recognition of OSCC cell lines and evaded phagocytosis of RAW 264.7 in vitro. The outstanding homogenous carcinoma targeting, and immune evading abilities were associated with excellent therapeutic performance of Asp8[H40- TPZ/IR780@(RBC-H)] on OSCC in vitro.

## Results and discussion

### Formulation and characterization of RBC-H vesicles

**Fig. 1:**
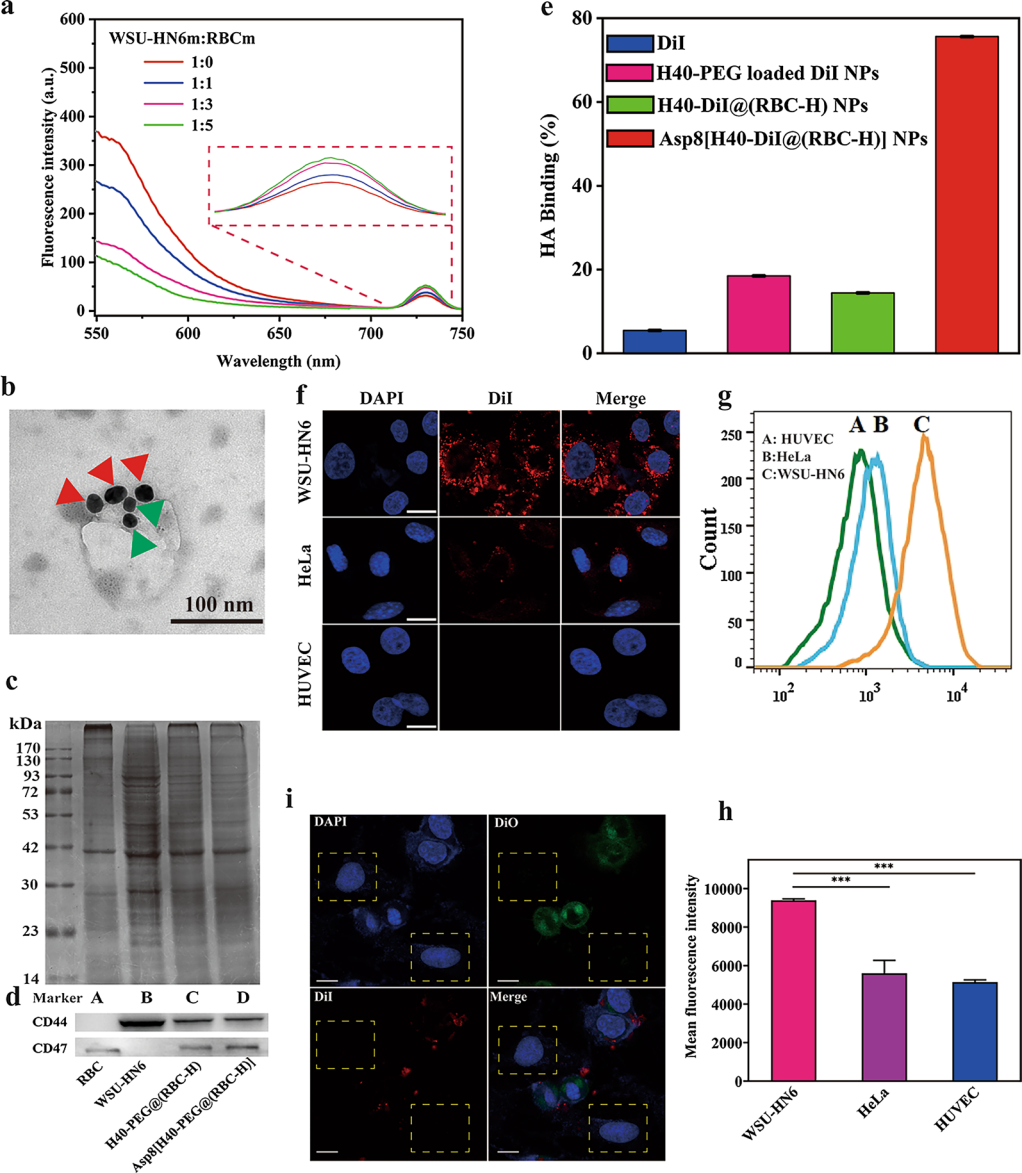
Characterization of hybrid membrane RBC-H and Asp8[H40-PEG@(RBC-H) NPs in vitro. **a** WSU-HN6 cell membrane doped with DiI and DiO and mixed with different mass ratios of RBCs membrane. The fluorescence recovery of the DiI was utilized to monitor the process of fusion. **b** Immunogold TEM images of RBC-H hybrid membrane vesicle probe for CD47 (the large gold NP, red arrow) and CD44 (the small gold NP, green arrow) (Scale bars = 100 nm). **c** SDS-PAGE analysis of protein retention (A: RBC membrane, B: WSU-HN6 membrane, C: H40-PEG@(RBC-H), D: Asp8[H40-PEG@(RBC-H) ). **d** Western blotting analysis of RBC membrane, WSU-HN6 membrane, H40-PEG@(RBC-H), and Asp8[H40-PEG@(RBC-H) for characteristic CD47 of the RBC membrane marker, and CD44 of the WSU-HN6 membrane marker. **e** HA binding ratio of Asp8[H40-DiI@(RBC-H)] NPs, H40-DiI@(RBC-H) NPs, H40-PEG loaded DiI NPs, and DiI after incubation with HA for 1 h at 37 ℃. **f** CLSM images of HeLa cells, HUVEC cells, and WSU-HN6 cells cultured with DiI dyed Asp8[H40-PEG@(RBC-H)] NPs. **g** Flow cytometric profiles and **h** mean fluorescence intensity of the WSU-HN6 cells, HeLa cells, and HUVEC cells incubated with DiI dyed Asp8[H40-PEG@(RBC-H)] NPs for 4 h at 37 ℃. **i** CLSM images of WSU-HN6 cells and HeLa cells cultured with DiI labeled Asp8[H40-PEG@(RBC-H)] NPs after co-incubated the both cell lines (green = WSU-HN6 cells membrane, bule = nucleus, and red = Asp8[H40-PEG@(RBC-H)] NPs, scale bar = 50 μm). Asterisks indicate a statistically significant difference between two groups (*p* < 0.05), *** *p* < 0.001

In this study, RBC-H hybrid membrane vesicles were fabricated though extrusion technology as previously reported [34]. To confirm the fusion, a FÖrster resonance energy transfer (FRET) pair dyes (DiI and DiO) were used to stain the WSU-HN6 cell membrane. Subsequently, as the RBC membrane weight increased, the changing FRET was recorded. Figure 1a shows that with increasing RBC membrane weight ratio, a recovery of fluorescence signal was discovered at 740 nm (from DiI). However, the fluorescence signal decreased at 550 nm (from DiO). These results indicate weakening of FRET process in the original DiO/DiI stained WSU-HN6 membrane due to interluding of the two cell membrane segments. Hybrid membranes were prepared at a membrane weight ratio (WSU-HN6: RBC) of 1:1 and were used to coat H40-PEG NPs when constructing the Asp8[H40-PEG@(RBC-H)] NPs. Additionally, confocal laser scanning florescence microscope (CLSM) was used to analyze cell membrane colocation. Before fusing, the WSU-HN6 cell membrane was labeled with DiO, and the RBC cell membrane was stained with DiI. And there was remarkable colocalization in RBC-H group, as revealed by the fluorescence signals derived from both DiI and DiO (Additional file 1: Figure S1). However, there was a negligible overlapping fluorescence signal in the mixture group of WSU-HN6 and RBC membranes without extrusion. In addition, immunogold labeled TEM imaging revealed analogous spherical nanoparticles, which contained two classes of colloidal golds with different sizes on the surface (Fig. 1b). These results indicated that the single Asp8[H40-PEG@(RBC-H)] nanoparticle exhibited both RBC and WSU-HN6 characteristic membrane protein markers.

### Formulation and characterization of Asp8[H40-PEG@(RBC-H)]

To evaluate the overall protein components of RBC membrane, WSU-HN6 membrane, H40-PEG@(RBC-H) NPs, and Asp8[H40-PEG@(RBC-H)] NPs, SDS-PAGE analysis was performed. As depicted in Fig. 1c, the protein profiles of Asp8[H40-PEG@(RBC-H)] NPs were comparable to those of H40-PEG@(RBC-H) NPs. Moreover, protein of RBC and WSU-HN6 membranes were found in Asp8[H40-PEG@(RBC-H)] NPs. These findings indicate the successfully fusion RBC-H hybrid membrane inherited proteins from RBC and WSU-HN6 membranes. To assess the inherited special protein markers, western blotting analysis was performed (Fig. 1d). CD47, which is expressed one most of protein markers in RBC membranes and can escape the macrophages phagocytosis, was detected on the RBC membrane, H40-PEG@(RBC-H) NPs, and Asp8[H40-PEG@(RBC-H)] NPs. CD44, which is overexpressed on WSU-HN6 and is involved in adherence and cell proliferation, was observed on the WSU-HN6 membrane, H40-PEG@(RBC-H) NPs, and Asp8[H40-PEG@(RBC-H)] NPs. These findings show that the RBC-H hybrid membrane was successfully fabricated. Furthermore, they suggest that biomimetic NPs can be constructed through hybrid membrane coating of H40-PEG NPs.

### Targeted characteristics study of Asp8[H40-PEG@(RBC-H)] in vitro

For the mandibula tissue invaded by oral cancer, the principal component of bone HA is exposed in erosion site [35]. It has been reported in some oligopeptides can reveal a strong affinity to mineral HA of bone tissue [36]. Due to the specific structure of aspartic, aspartic oligopeptide (Asp8) has been employed as bone-targeting ligand for drug delivery platform. To evaluate the bone targeting abilities of the biomimetic NPs, the HA binding experiment was performed in vitro. DiI was used as the fluorescence probe to label biomimetic NPs in this assay. As shown in Fig. 1e, approximately 78.0% of Asp8[H40-PEG@(RBC-H) NPs were bound to the hydroxyapatite power after incubation 1 h at 37 ℃. In contrast, for H40-PEG NPs and H40-PEG@(RBC-H) NPs, there were only less than 20.0% nanoparticles to bind the HA. These results indicate that the biomimetic NPs modified by Asp8 had desirable bone-targeting abilities, which provided the basis for the in vivo assay.

It has been reported that the cancer cell membrane-coated drug delivery platform has an excellent homotypic cancer targeting ability [37]. To verify the homotypic targeting ability of Asp8[H40-PEG@(RBC-H)] NPs, cellular uptake of DiI-labebled biomimetic NPs by WSU-HN6 cells, HeLa cells (human cervical cancer cells), and HUVE (human umbilical vein endothelial ) cells was evaluated by CLSM and flow cytometry. In this analysis, HUVECs were used as the control group. Figure 1f shows that WSU-HN6 cells exhibited significant red fluorescence intensities when compared to HeLa cells and HUVECs after incubation for 4 h at 37 ℃. To verify the specific self-recognition ability of biomimetic NPs, DiI-labebled biomimetic NPs were examined in co-incubated WSU-HN6 and HeLa cell lines by CLSM. As show in Fig. 1i, DiO (green fluorescence)-labeled WSU-HN6 cells were firstly co-cultured with HeLa cells. Then, the co-incubated cells were treated with DiI-labebled Asp8[H40-PEG@(RBC-H)] NPs (red fluorescence) for 4 h at 37 ℃. Negligible red fluorescence was found around the HeLa cells (in the yellow square). In contrast, there was a strong red fluorescence intracellular of WSU-HN6. The semiquantitative fluorescence intensity of DiI-labebled biomimetic NPs that targeted homologous WSU-HN6 cells were investigated by flow cytometry. Figure 1g, h show that mean fluorescence intensity of the WSU-HN6 group was higher approximately 1 and 2 folds than those of HeLa cells and HUVECs, respectively. The results indicate the specifical targeting ability of the biomimetic NPs toward WSU-HN6 cells. These findings show that the desirable self-recognition capability of Asp8[H40-PEG@(RBC-H)] NPs to source cells depend on membrane special protein molecules of WSU-HN6 to achieve homogenous cellular adhesion.

### Immune evasion study of Asp8[H40-PEG@(RBC-H)] in vitro

**Fig. 2:**
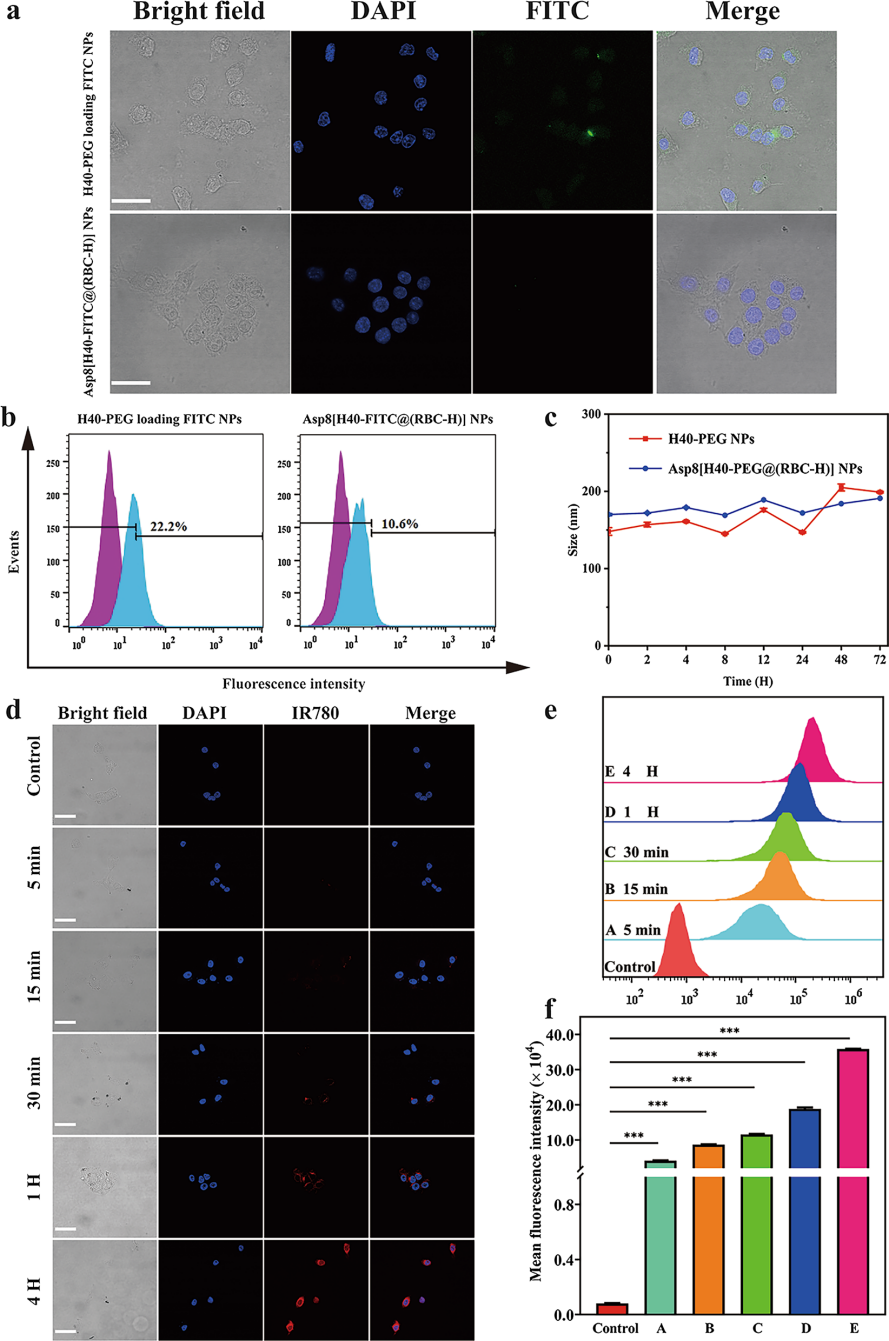
Characterization of biomimetic NPs. **a** CLSM images of RAW 264.7 cells treated with H40-PEG loading FITC NPs and Asp8[H40-FITC@(RBC-H)] NPs for 4 h at 37 ℃ (scale bar = 20 μm). **b** Fluorescence intensity detection by the flow cytometry. **c** Stability of Asp8[H40-PEG@(RBC-H)] NPs and H40-PEG NPs in PBS was detected by DLS. **d** CLSM images of WSU-HN6 cells treated with Asp8[H40-IR780@(RBC-H)] NPs for 5 min (**A**), 15 min (**B**), 30 min (**C**), 1 h (**D**), and 4 h (**E**) (scale bar = 25 μm). **e** The corresponding flow cytometry profiles of WSU-HN6 cells incubated with the biomimetic NPs for different time intervals. **f** The mean fluorescence intensity analysis after treated with the biomimetic NPs. *** *p* < 0.001

It is necessary to validate the immune evasion characteristic of Asp8[H40-PEG@(RBC-H)] NPs. And CLSM and flow cytometry were used to evaluate this ability by investigating the uptake of H40-PEG NPs and Asp8[H40-PEG@(RBC-H)] NPs by RWA 264.7 cells. As illustrated in Fig. 2a, the fluorescence intensity of Asp8[H40-FITC@(RBC-H)] NPs as detected by CLSM was significant weak compared to that of H40-PEG loaded FITC NPs group. Moreover, the relative fluorescence intensity of Asp8[H40-FITC@(RBC-H)] NPs group as shown by flow cytometry were approximately 10% less compared to that of the H40-PEG loaded FITC NPs (Fig. 2b), consistent with results of CLSM analysis (Additional file 1: Figure S2). These findings imply that the RBC-H hybrid membrane, which contained some special membrane proteins such as CD47, endowed the H40-PEG NPs with immune escape abilities to avoid macrophage phagocytosis. Therefore, Asp8[H40-FITC@(RBC-H)] NPs can avoid macrophage phagocytosis in vivo.

The stability of Asp8[H40-PEG@(RBC-H) NPs incubated with PBS for 3 days was evaluated by DLS. As shown in Fig. 2c, negligible changes were detected in the size of the biomimetic NPs compared to that of the bare nanoparticles. In addition, changes in hydrodynamic diameters of biomimetic NPs were analyzed before and after lyophilization by DLS. Additional file 1: Figure S3 shows that there were negligible size changes in these NPs prior to lyophilization and after lyophilization. Therefore, Asp8[H40-PEG@(RBC-H) NPs exhibited excellent stability properties.

### Cellular uptake of Asp8[H40-PEG@(RBC-H)]

The cellular uptake of Asp8[H40-PEG@(RBC-H)] NPs was assessed through CLSM and flow cytometry. Due to the red fluorescence property, IR780 was selected to label Asp8[H40-PEG@(RBC-H)] NPs while DAPI was used to stain the nucleus of WSU-HN6 cells. After incubated with IR780-labeled Asp8[H40-PEG@(RBC-H)] NPs at 37 ℃ for 5 min, 15 min, 30 min, 1 h, and 4 h, pretreated cells were observed by CLSM, respectively. Figure 2d shows that the red fluorescence signal was found in the cytoplasm after incubated with the IR780-labeled biomimetic NPs for 5 min. With the incubation time extended from 5 min to 1 h, the fluorescence intensity of the cells was gradually enhanced. After incubation for 4 h, the red fluorescence signal was observed in the nucleus of the cells. Therefore, the IR780-labeled biomimetic NPs could be efficiently internalized and IR780 could be released from the biomimetic NPs to reach the nucleus.

The internalized IR780-labeled biomimetic NPs were quantified by evaluating their fluorescence intensities. As shown in Fig. 2e, fluorescence intensity was observed after incubation for 5 min. The intracellular fluorescence intensity of IR780 increased with the extension of incubation time. After 4 h of incubation, mean fluorescence intensities of WSU-HN6 cells increased from 4.13 × 10^4^ to 35.87 × 10^4^ (Fig. 2f).Therefore, we postulated that the efficient internalization of Asp8[H40-PEG@(RBC-H)] NPs by WSU-HN6 cells may be attributed to the homotypic targeting abilities derived from RBC-H hybrid membrane as well as to the small hydrodynamic volume.

### Characterization of Asp8[H40-TPZ/IR780@(RBC-H)] NPs in vitro

Duo to the characteristic structure, such as considerable amounts of terminal functional groups, and branch structures, hyperbranched polymers (H40) have potential as drug delivery platforms. The TPZ and IR 780 were selected as antitumor and photothermal therapy drug models in this assay and were loaded into the H40-PEG NPs. After preparation of Asp8[H40-TPZ/IR780@(RBC-H) NPs according to the set theoretical DLC (5.0%), the final DLC of TPZ and IR780 were 2% and 2.92% measured via UV-vis spectrophotometry, respectively. Drug loading efficiency (DLE) of both drugs were also determined and found to be 52% and 60.90% for TPZ and IR780, respectively. These findings indicate that the hydrophobic drug (IR780) and hydrophilic drug (TPZ) can be co-loaded into inner nanocavities via oil-in-water emulsion solvent diffusion technology.

**Fig. 3:**
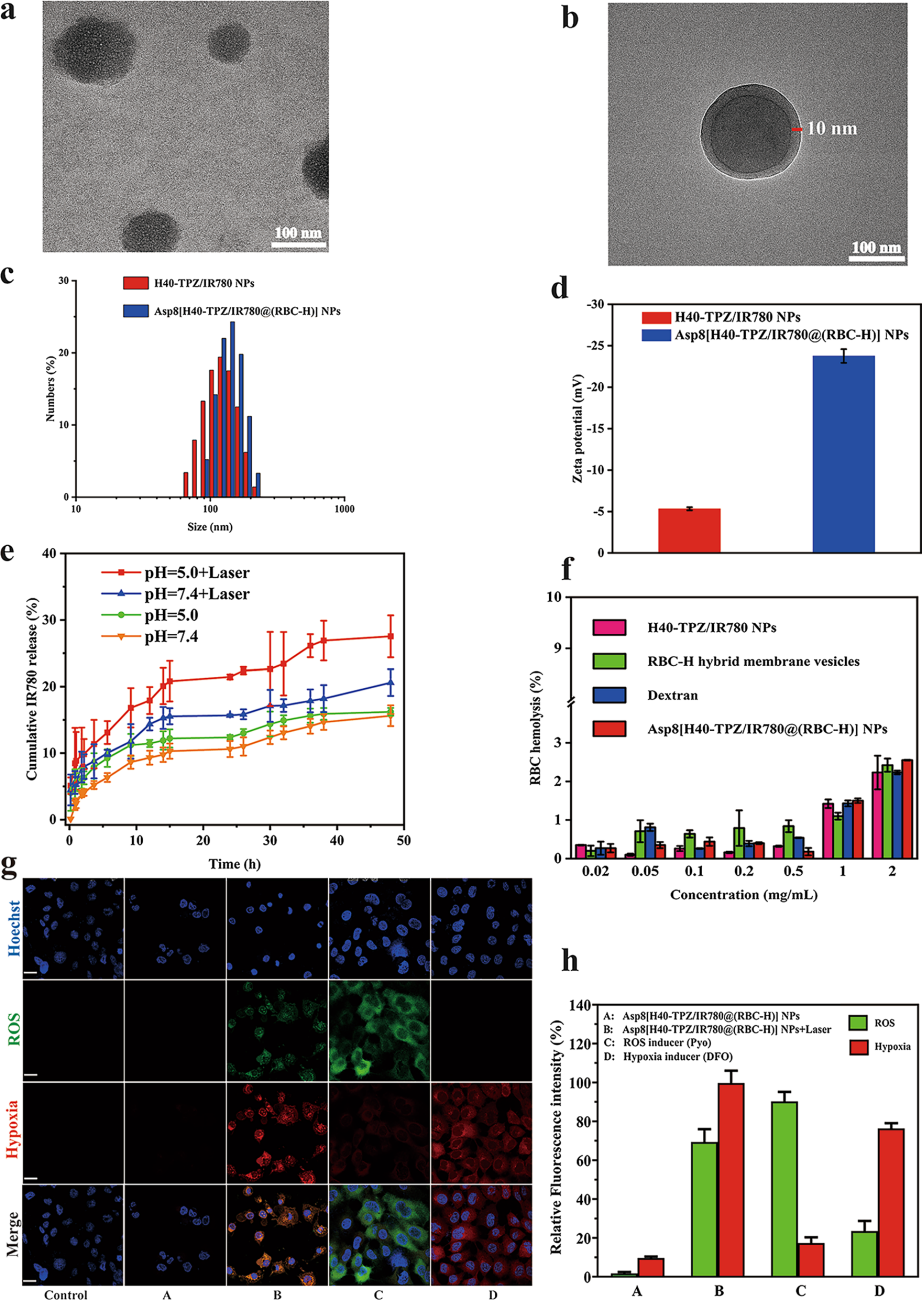
Characterization and drug release of Asp8[H40-TPZ/IR780@(RBC-H) NPs in vitro. **a** TEM images of H40-TPZ/IR780 NPs and **b** Asp8[H40-TPZ/IR780@(RBC-H) NP. **c** Average size and **d** surface zeta potential of bare H40-TPZ/IR780 NPs and Asp8[H40-TPZ/IR780@(RBC-H) NPs. The data are presented the average ± standard deviation (n = 3). **e** Release profiles of IR780 from the Asp8[H40-TPZ/IR780@(RBC-H)] NPs in different pH (5.0 and 7.4) buffer with or without laser irradiation. **f** Hemolysis assay of Asp8[H40-TPZ/IR780@(RBC-H)] NPs compared with RBC-H hybrid membrane vesicles, H40-TPZ/IR780 NPs, and dextran. 1 % Triton X-100 was used as a 100% hemolytic value. **g** CLSM and **h** relative fluorescence intensity analysis of ROS generation and hypoxia of WSU-HN6 incubated with PBS, Asp8[H40-TPZ/IR780@(RBC-H)] NPs without (**A**) and with (**B**) laser irradiation, Pyo (**C**), and DFO (**D**) for different time using Image J (scale bar = 25 μm)

Size is an important characteristic for a nanoparticle to be an effective drug delivery vehicle. Apart from the RBC-H hybrid membrane, small-sized NPs (< 200 nm) are important for avoiding immune recognition and clearing. Furthermore, these NPs can enhance passive targeting of tumor tissue [38]. To evaluate the properties of Asp8[H40-TPZ/IR780@(RBC-H) NPs, TEM and DLS were used. As shown in Fig. 3a, b, TEM analysis revealed that Asp8[H40-TPZ/IR780@(RBC-H) NPs exhibited spherical morphologies with a characteristic core-shell structure. The average diameter of biomimetic NPs was approximately 150 nm, which increased by about 10 nm over that of bare H40-TPZ/IR780 NPs. This was attributed to coating with the RBC-H hybrid membrane. Thickness of the out RBC-H hybrid membrane was approximately 10 nm (Fig. 3b). The hydrodynamic diameter and ζ potential of biomimetic NPs were further analyzed by DLS. As illustrated in Fig. 3c, the size of this resulting biomimetic NPs increased to 170.45 nm after coating with the RBC-H hybrid membrane, while that of the bare H40-TPZ/IR780 NPs was approximately 149.83 nm. In addition, surface zeta potential changed from − 5.32 to − 23.76 mV after the bare H40-TPZ/IR780 NPs had been camouflaged with RBC-H hybrid membrane (Fig. 3d). These findings indicate shielding of negative H40-TPZ/IR780 NPs by the more negative outer hybrid membrane layers.

### IR780 and TPZ release in vitro

The release profile of Asp8[H40-TPZ/IR780@(RBC-H) NPs in vitro was assessed through dialysis in different pH buffer solutions (pH=7.4 and 5.0) with or without laser irradiation (808 nm, 1.0 W cm^−2^, 5 min) at 37 ℃. Figure 3e and Additional file 1: Figure S4 show that without laser irradiation, IR780 and TPZ cumulative release rates in a medium with pH 7.4 within 48 h was about 15.63% and 27.37%, respectively. At pH 5.0, release rates were 16.22% and 43.98%, respectively. For the medium of pH 7.4 under 5 min laser irradiation within 48 h, there were no significant changes in release profiles of IR780 and TPZ. In contrast, with the laser irradiation for 5 min, cumulative release rates of IR780 and TPZ in the buffer with pH 5.0 within 48 h were 27.54% and 46.74%, respectively. These findings imply that weak acidic surroundings and local hyperthermia can damage the outer cell membrane and enhance drug diffusion from the interior to the microenvironment. Furthermore, the slow release nature of the biomimetic reveals that both drugs can maintain an effective dose over a relatively long period.

### Hemolysis analysis of Asp8[H40-TPZ/IR780@(RBC-H)] in vitro

Hemocompatibility is important for application of drug delivery vehicles in vivo [39]. To evaluate the biocompatibility of biomimetic NPs in physiological conditions, the hemolysis assay was performed in vitro [40]. The 1.0% v/v Triton X-100 was employed as 100 % hemolysis while Dextran was used as the reference group in this assay. As shown in Fig. 3f, there were no significant difference in hemoglobin release by Asp8[H40-TPZ/IR780@(RBC-H) NPs, RBC-H hybrid membrane vesicles, and H40-TPZ/IR780 NPs when compared to hemoglobin release by Dextran. The released hemoglobin in these groups was also less than 3.0% even in the concentration up to 2.0 mg/mL after incubated with RBC solution for 1 h. Therefore, Asp8[H40-TPZ/IR780@(RBC-H)] NPs did not exhibit a concentration-dependent hemoglobin release characteristic. Less than 10% hemolysis is regarded as safe for a drug delivery system. From our findings, the biomimetic NPs had excellent biosafety properties.

### ROS/hypoxia analysis of Asp8[H40-TPZ/IR780@(RBC-H)] in vitro

To determine if Asp8[H40-TPZ/IR780@(RBC-H)] NPs-mediated lighted-triggered intracellular reactive oxygen species (ROS) production and hypoxia levels, CLSM was employed to observe the fluorescence intensity of ROS/hypoxia detection probes. As shown in Fig. 3g, Asp8[H40-TPZ/IR780@(RBC-H)] NPs with 808 nm laser irradiation for 5 min (1.0 W cm^−2^) exhibited significant levels of hypoxia (red) and ROS (green) compared to the PBS, Asp8[H40-TPZ/IR780@(RBC-H)] NPs without laser irradiation, ROS inducer, and hypoxia inducer, respectively. Moreover, relative fluorescence intensity analysis confirmed the ability of Asp8[H40-TPZ/IR780@(RBC-H)] NPs to mediate lighted-triggered intracellular ROS generation and hypoxia levels (Fig. 3h).

### Anticancer assay in vitro

It is important that biomimetic NPs produce negligible or low toxicity to normal tissues [41]. Therefore, we evaluated the cytotoxic properties of Asp8[H40-PEG@(RBC-H)] NPs against both HUVEC and WSU-HN6 cells without laser irradiation. Additional file 1: Figure S5, S6 showed that cytotoxic effects of the biomimetic vehicle against both cells were insignificant. Cell viabilities of all groups were approximately 80% even though the concentration of NPs was up to 40.0 µg mL^−1^ after 72 h incubation. Therefore, the biomimetic drug delivery vehicles exhibited low cytotoxicity effects to HUVEC and WSU-HN6 cells.

**Fig. 4:**
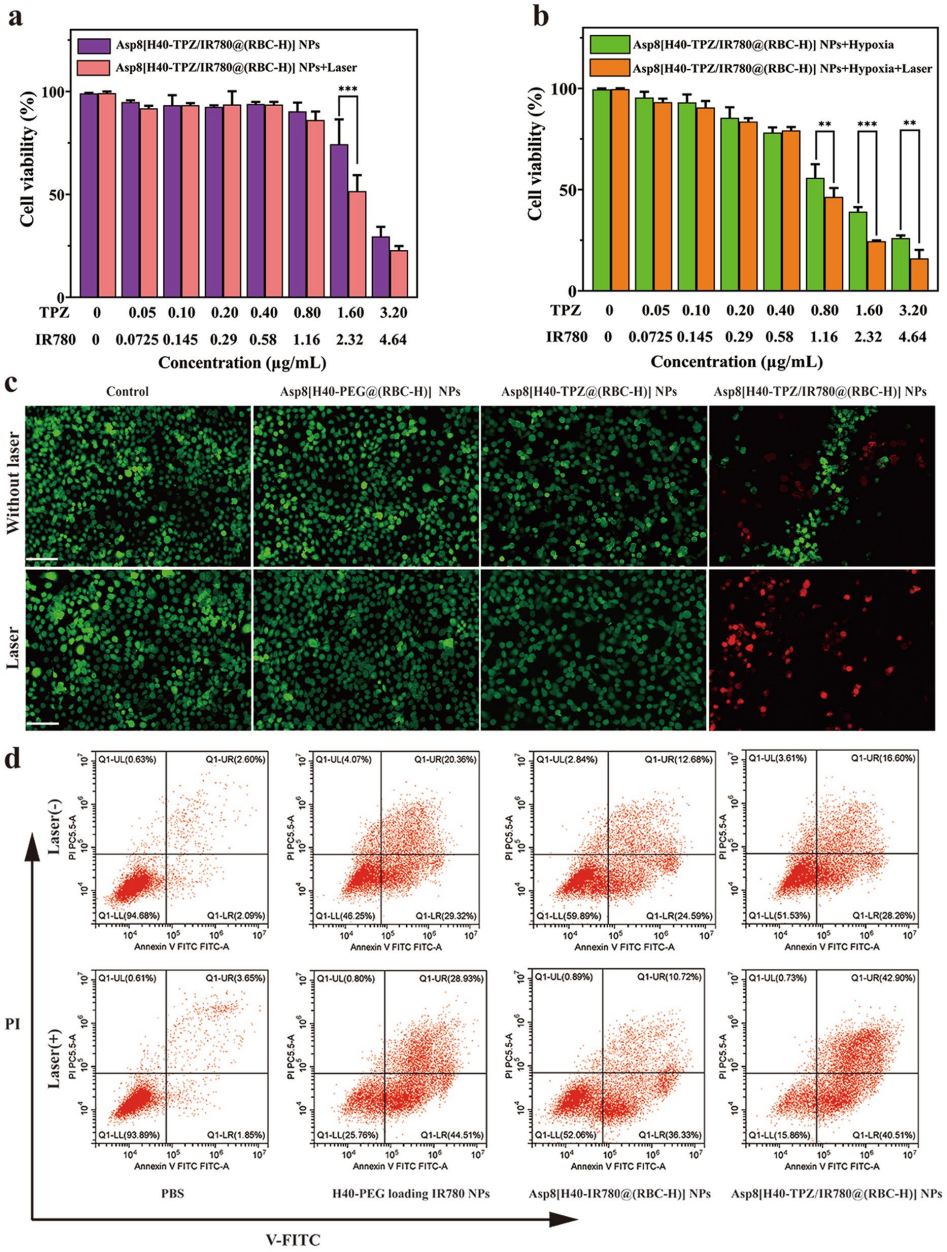
Anticancer efficacy of Asp8[H40-TPZ/IR780@(RBC-H) NPs in vitro. **a** The viability of WSU-HN6 cells treated with different concentrations of Asp8[H40-TPZ/IR780@(RBC-H)] NPs with/without laser irradiation under normoxic or **b** hypoxia environment. And the cells incubated without nanoparticles were utilized as the control. **c** The fluorescent microscopy images of live/dead staining of WSU-HN6 cells incubated with different formulations with/without laser irradiation (scale bar = 50 μm). **d** The apoptosis analysis by Annexin V-FITC/PI staining of WSU-HN6 cells after treated with PBS, H40-PEG loading IR780 NPs, Asp8[H40-IR780@(RBC-H)] NPs, and Asp8[H40-TPZ/IR780@(RBC-H)] NPs without and with laser irradiation. All data are exhibited as the average ± standard deviation (n=3). **p < 0.01 and *** *p* < 0.001

Cancer cell proliferation inhibitory properties of Asp8[H40-TPZ/IR780@(RBC-H)] NPs were assessed through the CCK-8 kit against WSU-HN6 cells in vitro. WSU-HN6 cells were treated with Asp8[H40-TPZ/IR780@(RBC-H)] NPs at different TPZ/IR780 concentration with/without laser irradiation (808 nm, 1.0 W cm^−2^, 5 min) for 24 h in a normoxic and hypoxic environment, respectively. Under normoxic condition, all Asp8[H40-TPZ/IR780@(RBC-H)] NPs exhibited weak cell cytotoxicity with/without laser irradiation (Fig. 4a). The viability of WSU-HN6 cells decreased only to approximately 51.61 % in the laser irradiation group even if the TPZ and IR780 concentration increased to 1.60 µg mL^−1^ and 2.32 µg mL^−1^, respectively. In contrast, under hypoxic environment, Asp8[H40-TPZ/IR780@(RBC-H)] NPs showed strong cytotoxicity (Fig. 4b). The cell viability could decrease to approximately 39.11% without laser irradiation under the same concentration of TPZ and IR780. As shown in Fig. 4b, compared to cell viability of Asp8[H40-IR780@(RBC-H)] NPs without laser irradiation, that of the laser irradiation group declined to the lowest (16.03%) when the biomimetic NPs increased to the highest concentration (TPZ: 3.20 µg mL^−1^, IR780: 4.64 µg mL^−1^). These findings indicated that the synergetic effect of PTT, which induced bioreductive chemotherapy in the hypoxic microenvironment, enhanced cytotoxicity of the Asp8[H40-IR780@(RBC-H)] NPs. Therefore, the combination loading TPZ and IR780 provides excellent therapeutic efficacies in hypoxia environment. Furthermore, Asp8[H40-TPZ/IR780@(RBC-H)] NPs achieved a favorable synergistic anticancer effect through phototherapy and hypoxia-activated chemotherapy. Moreover, live and dead staining was performed to verify proliferation inhibition effects on WSU-HN6 cells. Cells in green are live cells while those in red are dead cells. As shown in Fig. 4c, green fluorescence was observed in each group without laser irradiation. Comparable results were detected in groups of Asp8[H40-PEG@(RBC-H)] NPs and Asp8[H40-TPZ@(RBC-H)] NPs with the 808 nm laser irradiation for 5 min (1.0 W cm^−2^). A little red fluorescence was found in group of Asp8[H40-TPZ/IR780@(RBC-H)] NPs without laser irradiation. In contrast, Asp8[H40-TPZ/IR780@(RBC-H)] NPs exhibited a significant red fluorescence signal upon laser irradiation of the same intensity. The strong red fluorescence of Asp8[H40-TPZ/IR780@(RBC-H)] NPs with laser irradiation group imply a high photothermal effect of biomimetic NPs. Furthermore, the hypoxia-activated chemotherapy drug (TPZ) also played an important role during the synergistic therapy. Therefore, the biomimetic NPs have outstanding synergistic therapeutic effects.

Cell apoptosis induced by Asp8[H40-TPZ/IR780@(RBC-H)] NPs with laser irradiation was analyzed though Annexin V-FITC/PI assay. As illustrated in Fig. 4d, WSU-HN6 cells incubated with H40-PEG loading IR780 NPs, Asp8[H40-IR780@(RBC-H)] NPs, and Asp8[H40-TPZ/IR780@(RBC-H)] NPs without laser irradiation exhibited a moderate number of apoptotic cells. In contrast, corresponding groups revealed a large number of apoptotic cells upon laser irradiation (808 nm, 1.0 W cm^−2^, 5 min). The maximum of approximately 84.41% apoptotic cells, which included both early (40.51%) and late (42.90%) apoptotic cells, were observed in Asp8[H40-TPZ/IR780@(RBC-H)] NPs with laser irradiation group. These results indicate that the Asp8[H40-TPZ/IR780@(RBC-H)] NPs can produce significant cytotoxic effects upon laser irradiation.

### Photothermal assay in vitro **and** in vivo

**Fig. 5:**
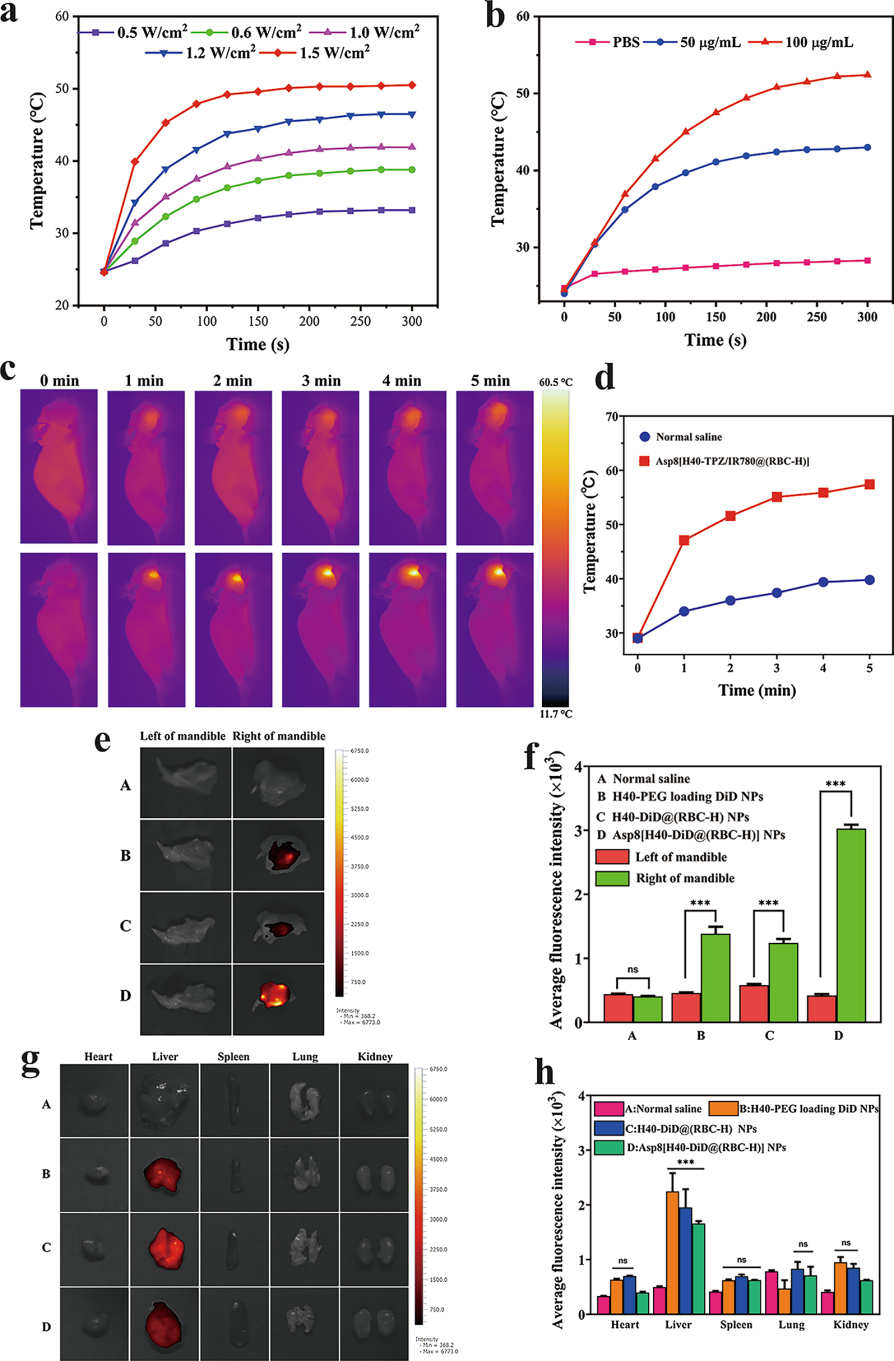
Photothermal profiles and biodistribution of the biomimetic NPs. **a** The temperature rising profiles of Asp8[H40-TPZ/IR780@(RBC-H)] NPs (IR780: 50 µg mL^−1^) subjected to the irradiation of 808 nm laser with different power densities for 5 min. **b** The temperature variation curves of Asp8[H40-TPZ/IR780@(RBC-H)] NPs with different concentrations of IR780 and PBS irradiated with laser. **c** Infrared imaging photos and **d** temperature change curves of tumor-bearing mice intravenously injected with normal saline and Asp8[H40-TPZ/IR780@(RBC-H)] NPs with laser irradiation at the power density of 1.0 W cm^−2^ for 5 min. **e** *Ex vivo* the fluorescence images of the separated mandibula and **g** major organs of tumor-bearing mice at 8 h after injection with normal saline (A), H40-PEG loading DiD NPs (B), H40-DiD@(RBC-H) NPs (C), and Asp8[H40-DiD@(RBC-H)] NPs (D). **f** Average DiD fluorescence intensities of the separated mandibula and **h** major organs at 8 h with normal saline and different DiD-labeled NPs. The data are presented the average ± standard deviation (n = 3). *** *p* < 0.001, ns indicates no significant difference

The photothermal performance of Asp8[H40-TPZ/IR780@(RBC-H)] NPs in vitro were evaluated through an infrared camera. Figure 5a shows that Asp8[H40-TPZ/IR780@(RBC-H)] NPs (IR780: 50 µg mL^−1^) in solution were irradiated with an 808 nm laser at different power densities for 5 min. With extension of irradiation time, the temperature of the biomimetic NPs solution rapidly increased within the first 180s, reaching the maximum at 50.53 ℃ after 808 nm laser irradiation at 1.5 W cm^−2^ for 5 min. Temperatures of different concentrations of these biomimetic NPs were also evaluated under 808 nm laser irradiation at 1.0 W cm^−2^ for 5 min (Fig. 5b). Compared to the increasing temperature in the low concentration group (IR780: 50 µg mL^−1^), the temperature of the group (IR780: 100 µg mL^−1^) rapidly increased. Therefore, photothermal transformation characteristics of biomimetic NPs were time-dependent, power-dependent, and concentration-dependent. To visually observe temperature changes of biomimetic NPs, IR thermal images were captured for 5 min at predetermined time intervals. For Asp8[H40-TPZ/IR780@(RBC-H)] NPs (IR780: 100 µg mL^−1^), pseudo-color signals were gradually enhanced with extending irradiation time (808 nm, 1.0 W cm^−2^). However, there were no significant changes for the normal saline, 10% FBS, and PBS groups under the same irradiation conditions (Additional file 1: Figure S7). Therefore, Asp8[H40-TPZ/IR780@(RBC-H)] NPs is a potential agent for photothermal therapy.

It was confirmed that Asp8[H40-TPZ/IR780@(RBC-H)] NPs have excellent photothermal conversion abilities in vitro. To evaluate photothermal features of the biomimetic NPs in vivo, WSU-HN6 tumor-bearing mice were used in this analysis. For the mice injected with Asp8[H40-TPZ/IR780@(RBC-H)] NPs, pseudo-color at the tumor site gradually deepened with increasing laser (808 nm, 1.0 W cm^−2^) irradiation time according to Fig. 5c. Moreover, temperature at the location rapidly increased to 57.42 ℃ within 5 min as shown in Fig. 5d. Conversely, for the mice with normal saline injection, changes in pseudo-color at the tumor site was insignificant with increasing laser irradiation time. And the temperature of the tumor site was only increased to approximately 39.82 ℃. These findings show that biomimetic NPs can provide a desirable temperature for photothermal therapy.

### Biodistribution assay in vivo

Dual-targeted effects of biomimetic nanoparticles in vivo were further verified using a WSU-HN6 female nude mice tumor model. In this experiment, the normal saline group was used as negative control. At 8 h after tail vein injection with various DiD-loading NPs, mandibula and major organs were dissected from the nude mice and detected using a VISQUE imager. Compared to DiD fluorescence signal intensity in the left mandibula, the right tumor-bearing mandibula exhibited significant fluorescence signal (Fig. 5e). As revealed in Fig. 5f, the average fluorescence intensity analysis confirmed the above results. Additionally, average fluorescence intensity in group D was highest when compared to group A, B, and C. Moreover, quantitative signal intensity of DiD in group D was approximately twofold higher than that of group B or C. Therefore, the biomimetic NPs can efficiently cluster at local sites of bone invasion through Asp8-mediated bone-targeting. In addition, efficient clustering could also be due to homogenous targeting abilities mediated by the WSU-HN6 cell membrane segment. As shown in Fig. 5g, most of the DiD fluorescence signals were detected in the liver. Only a few fluorescence signals were detected in other organs. Compared to groups B and C, group D exhibited the lowest signals in the liver. The average fluorescence intensity in the liver was approximately onefold lower than that of the group B (Fig. 5h). Moreover, there was no statistically significant difference in fluorescence signals in the heart, spleen, lung, and kidney for groups B, C, and D, respectively. This result suggests that the RBC cell membrane of the hybrid membrane can enable H40-PEG NPs to escape recognizing and clearing by the reticuloendothelial system (RES). Therefore, the Asp8[H40-DiD@(RBC-H)] NPs exhibited dual-targeting characters in vitro and in vivo.

### Anticancer activity in vivo

**Fig. 6:**
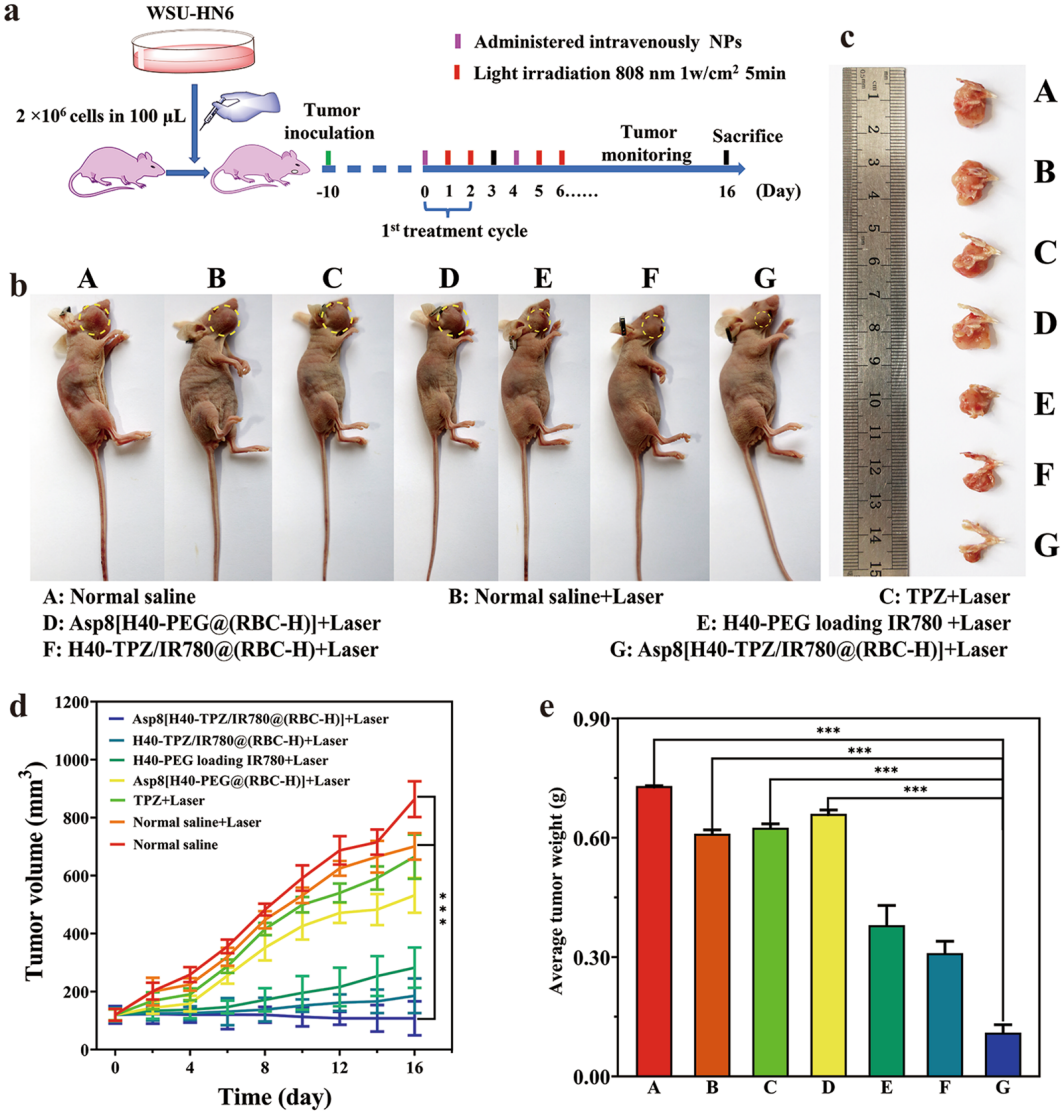
Anticancer efficacy of Asp8[H40-TPZ/IR780@(RBC-H)] NPs in vivo. **a** Schematic illustration of therapeutic scheme on bone invasion nude mice model. **b** Photos of WSU-HN6 carcinoma-bearing nude mice. **c** Tumor photos from each group ex vivo (A: Normal saline, B: Normal saline + Laser, C: TPZ + Laser, D: Asp8[H40-PEG@(RBC-H)] + Laser, E: H40-PEG loading IR780 + Laser, F: H40-TPZ/IR780@(RBC-H) + Laser, and G: Asp8[H40-TPZ/IR780@(RBC-H)] + Laser). **d** Tumor growth curves of WSU-HN6 tumor-bearing nude mice subjected to various therapy and **e** the average tumor weight after dissection from each group (n = 5, mean ± SD). **p < 0.01 and *** *p* < 0.001

The mandibular invasion mouse model was used to assess synergistic photothermal and chemotherapy effects of Asp8[H40-TPZ/IR780@(RBC-H)] NPs. After successful of the bone invasion model, the WSU-HN6-tumor-bearing nude mice were randomly assigned into 7 groups (n = 5). Mice were treated with various formulations with or without laser irradiation (808 nm, 1.0 W cm^−2^, 5 min) as revealed in Fig. 6a. Compared to normal saline and normal saline with laser (control groups), neither TPZ nor Asp8[H40-PEG@(RBC-H)] exhibited significant anticancer effects upon laser irradiation (Fig. 6b–d). As shown in Fig. 6b–d, carcinoma growth inhibition efficiency of H40-TPZ/IR780@(RBC-H)] NPs was better than that of H40-PEG loading IR780 NPs upon laser irradiation. These findings indicate that H40-PEG loaded IR780 bare nanoparticles can be quickly cleared by the RES. Compared to all the above groups, Asp8[H40-TPZ/IR780@(RBC-H)] NPs exhibited the most excellent tumor-growth inhibitory effect upon laser irradiation. This could be attributed to the hybrid membrane coating and the Asp8 bone-targeting ligand of biomimetic NPs. As shown in Fig. 6e, the average tumor weight in the Asp8[H40-TPZ/IR780@(RBC-H)] group with laser was approximately threefold lighter than that of the control group (*p* < 0.001). Additionally, the excellent carcinoma therapeutic efficiency of the dual-targeting biomimetic NPs revealed that chemotherapeutic effects of TPZ can be synergistically enhanced by IR780-based photothermal therapy. Therefore, Asp8[H40-TPZ/IR780@(RBC-H)] NPs can precisely localize bone invasion sites to exert anticancer effects.

**Fig. 7:**
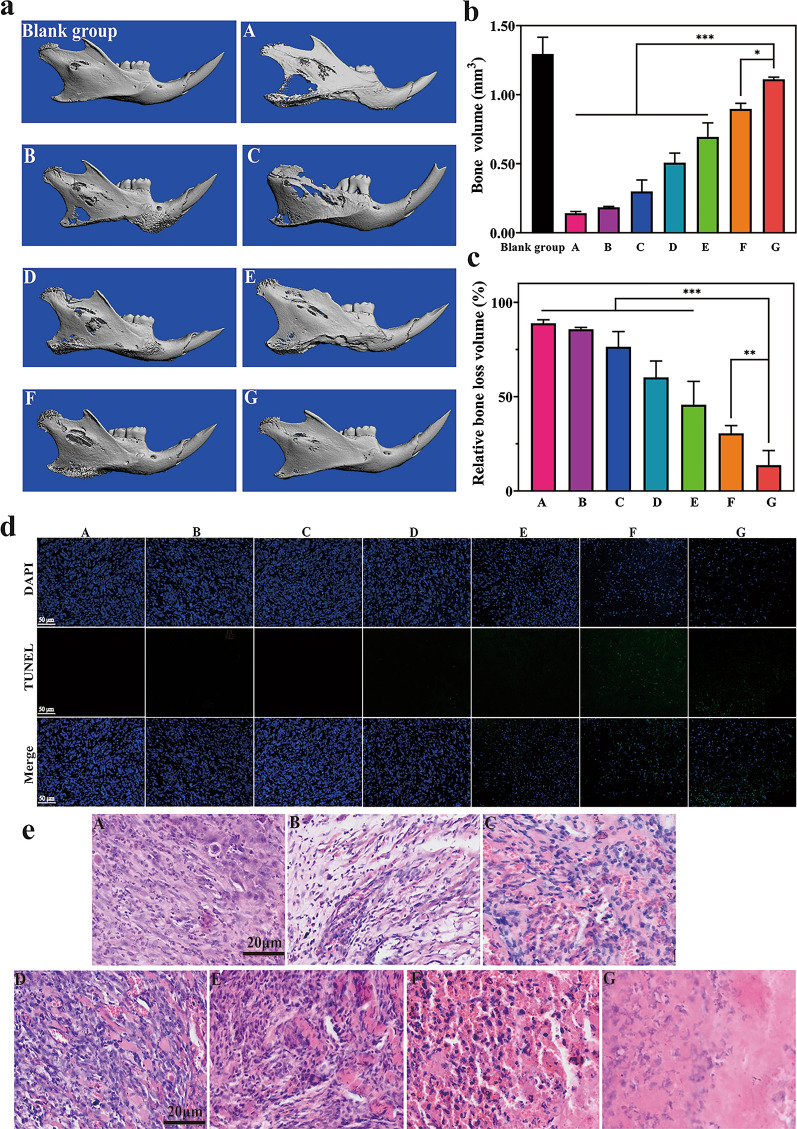
Histological analysis after the formulations therapy. **a** Micro-CT 3D reconstruction images of right part of mandibular from tumor-bearing mice after therapy. **b** Bone volume of 3D architecture for the right part of mandibular. **c** Relative bone loss volume of right part of mandibular from tumor-bearing mice after various formulations treatment. **d** TUNEL staining images of tumor tissues with various formulations treatment (scale bar = 50 μm). **e** H&E staining images of tumor tissues harvested from the tumor-bearing mice with different therapy. (A: Normal saline, B: Normal saline + Laser, C: TPZ + Laser, D: Asp8[H40-PEG@(RBC-H)] + Laser, E: H40-PEG loading IR780 + Laser, F: H40-TPZ/IR780@(RBC-H) + Laser, and G: Asp8[H40-TPZ/IR780@(RBC-H)] + Laser). **p < 0.01 and *** *p* < 0.001

To assess the degree of bone destruction by oral carcinoma, the right mandibula were isolated from the sacrificed tumor-bearing mice with different therapies. Compared to the bone resorption in the mandibular angle of mice treated with saline, saline + laser, PTZ + laser, and Asp8[H40-PEG@(RBC-H)] + laser, there was less destruction in the mandibula of mice administered with H40-PEG loaded IR780 NPs, H40-TPZ/IR780@(RBC-H)] NPs, and Asp8[H40-TPZ/IR780@(RBC-H)] NPs upon laser irradiation (Fig. 7a). Notably, mice administered with Asp8[H40-TPZ/IR780@(RBC-H)] NPs and laser irradiation retained an intact mandibula shape compared to the control group. Therefore, Asp8[H40-TPZ/IR780@(RBC-H)] NPs can effectively prevent bone destruction due to oral carcinoma through the bone/tumor-targeting ligand of these vehicles. Moreover, the 3D architecture parameters of the right mandibula such as bone volume and relative bone loss volume were further analyzed. Compared with the blank group, the bone volume in normal saline, normal saline + Laser, TPZ + Laser, Asp8[H40-PEG@(RBC-H)] + Laser, H40-PEG loading IR780 + Laser, and H40-TPZ/IR780@(RBC-H)] + Laser groups was significantly decreased, while it in Asp8[H40-TPZ/IR780@(RBC-H)] + Laser group was a slight but not significant decrease (Fig. 7b). The results were also confirmed by the relative bone loss volume analysis exhibited in Fig. 7c. These data confirm that Asp8[H40-TPZ/IR780@(RBC-H)] NPs could well protect the microstructure of bone from damage by invasion of carcinoma.

Furthermore, tumor histological assays were performed to evaluate changes at the cellular level. As shown in Fig. 7d, TUNEL staining analysis revealed that cancer cells were damaged after been administered with H40-PEG loaded IR780 NPs + laser, H40-TPZ/IR780@(RBC-H)] NPs + laser, and Asp8[H40-TPZ/IR780@(RBC-H)] NPs + laser, respectively. A substantial number of apoptotic cancer cells were detected in the Asp8[H40-TPZ/IR780@(RBC-H)] NPs + laser group compared to other groups. Hematoxylin and eosin (H&E) staining confirmed these findings. As revealed in Fig. 7e, the number of apoptotic cells in the Asp8[H40-TPZ/IR780@(RBC-H)] with laser irradiation is significantly more than that of other groups. Tumor tissue in the Asp8[H40-TPZ/IR780@(RBC-H)] NPs + laser group exhibited striking nuclear deformation in H&E staining, while other groups had less nuclear deformation, indicating that the tumor tissue in the photochemotherapy group had significant cancer cell necrosis.

Taken together, results of synergistic therapy in vivo indicated that Asp8[H40-TPZ/IR780@(RBC-H)] NPs exert the extraordinary anticancer effects in oral cancer invasion sites.

### Biosafety in vivo

**Fig. 8:**
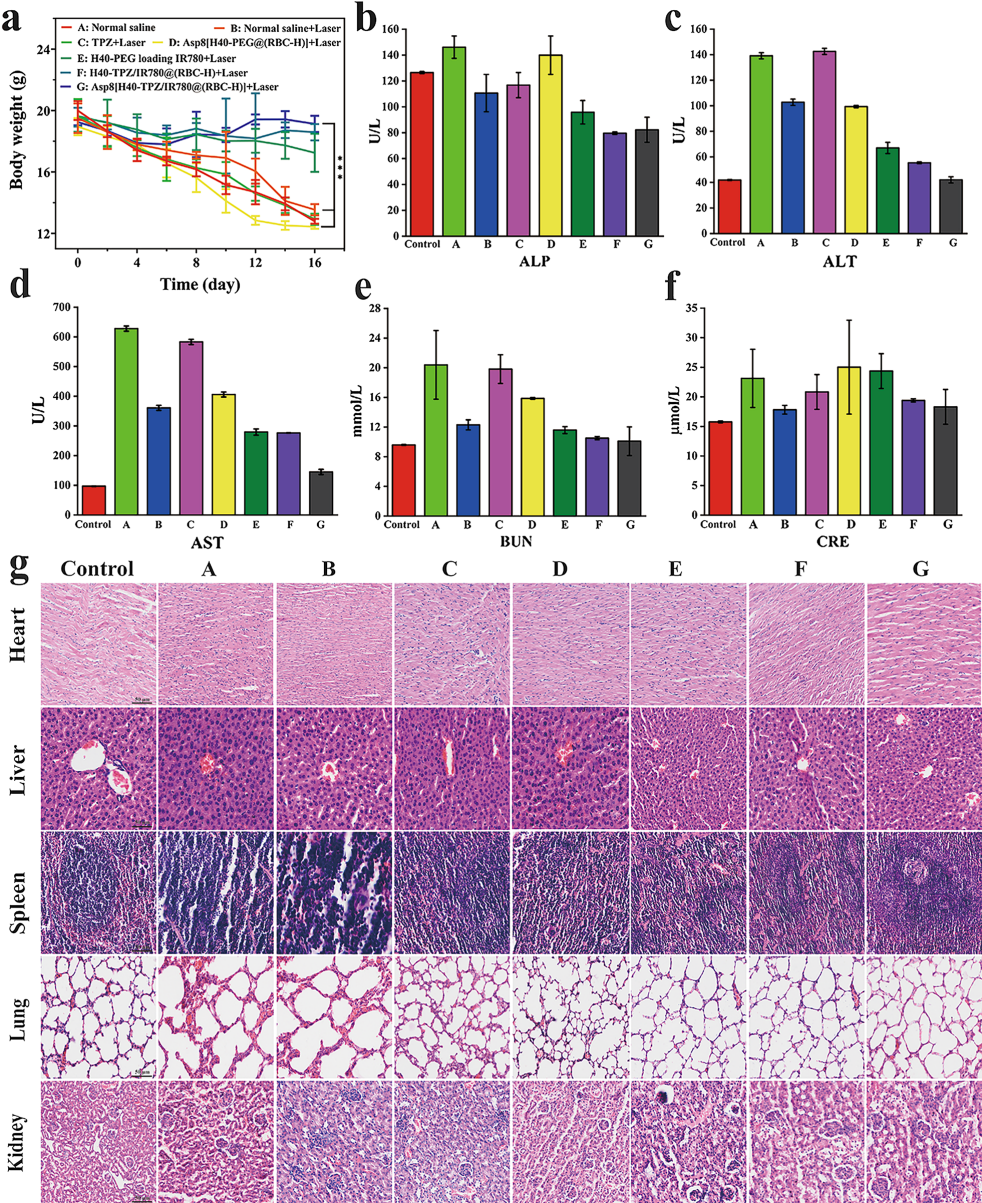
Preliminary biosafety assay of Asp8[H40-TPZ/IR780@(RBC-H)] NPs. **a** The body weight change curves of nude mice bearing WSU-HN6 tumor after various treatment (n=5, mean ± SD). **b–d** Main blood biochemical parameters of liver function including ALP, ALT, and AST. **e**, **f** Main biochemical parameters of kidney function including BUN and CRE. **g** H&E staining slices of major organs including heart, liver, spleen, lung, and kidney from each group. The mice without any therapy were as a blank group (scale bar = 20 μm). (A: Normal saline, B: Normal saline + Laser, C: TPZ + Laser, D: Asp8[H40-PEG@(RBC-H)] + Laser, E: H40-PEG loading IR780 + Laser, F: H40-TPZ/IR780@(RBC-H) + Laser, and G: Asp8[H40-TPZ/IR780@(RBC-H)] + Laser). *** *p* < 0.001

A low toxic side effect, especially to major organs, is a crucial index for evaluating the safety of nanoparticles for anticancer therapy [42, 43]. To some extent, body weight fluctuation is an indicator of toxic effects of formulations in vivo [44]. As shown in Fig. 8a, the decrease in body weights for mice in the Asp8[H40-TPZ/IR780@(RBC-H)] NPs therapeutic group was not significant when compared to other groups. The body weight loss was attributed to special location of tumors and effects of anticancer agents. With carcinoma growth, mice gradually developed difficulties in mouth opening, which seriously affected eating. Blood biochemical analyses were also performed to assess the safety of NPs on the 16th day after therapy. As depicted in Fig. 8b–d, levels for biochemical parameters of liver functions, such as alkaline phosphatase (ALP), alanine transaminase (ALT), and aspartate transaminase (AST) for mice in the Asp8[H40-TPZ/IR780@(RBC-H)] + laser group were not significantly different when compared to those of the control group. In addition, levels of kidney function markers, such as blood urine nitrogen (BUN) and creatinine (CRE), were comparable to those of the control group (Fig. 8e, f). As shown in Fig. 8c–f, there was significant damage to the liver and renal function in the normal saline group as compared with the control. A possible reason for this was that the mice developed long-term food intake difficulties, resulting in damage to the liver and renal function in turn. The results were consistent with the changes of body weight. H&E staining analysis was also performed to verify the biosafety of this formulation. As illustrated in Fig. 8g, there were significant alterations in tissue structures of the liver, spleen, and lungs in mice groups treated with normal saline, normal saline + laser, TPZ + laser, and Asp8[H40-PEG@(RBC-H)] + laser when compared to the control group. However, histological structure changes for major organs in the Asp8[H40-TPZ/IR780@(RBC-H)] + laser group compared to the control group were not significant. These findings indicate that biomimetic NPs had extremely low toxicity and excellent biosafety properties. Therefore, dual targeting drug delivery vehicles may be of potential clinical utilities for oral cancer bone invasion.

## Conclusions

We successfully fused the red blood cell membranes with OSCC cell line WSU-HN6 cell membranes and prepared an erythrocyte-cancer hybrid membrane-camouflaged H40-TPZ/IR780 NPs for targeting oral cancer bone invasion. Compared to erythrocyte and WSU-HN6 cells, hybrid RBC-H membrane surfaces contained the same membrane proteins. Due to Asp8 on the surface of the hybrid membrane, inner nanoparticles exhibited bone targeting abilities. Moreover, through the WSU-HN6 cell membrane, nanoparticles were endowed with the ability to target homologous carcinoma cells. And the PEG-TPZ/IR780 nanoparticles could be actively delivered to cancer cells located in bone invasion sites by this surface biofunctionalization. Moreover, Asp8[H40-TPZ/IR780@(RBC-H)] NPs exhibited excellent immune escape properties, which were attributed to erythrocyte membrane coating.

Under excitation of 808 nm laser, IR780 loaded in Asp8[H40-TPZ/IR780@(RBC-H)] NPs generated heat, while TPZ was also released gradually at the same time. Therefore, the synergistic effect of photothermal and hypoxia-activated chemotherapy was enhanced for cancer in bone invasion sites without obvious injuries to main organs. Therefore, synthesized nanoparticles can be conferred with multiple and additional biological functions by the hybrid membrane coating technology. Therefore, the dual targeting biomimetic drug delivery nanoplatform can be applied for precise therapy of malignancy in bones.

## Experimental section

### Materials and reagents

IR780 was obtained from Sigma-Aldrich and used as received. TPZ was obtained from MedChemExpress and used as received. All other chemicals were purchased from Chongqing Chuandong Chemical (group) CO., Ltd. and used without further purification. All chemicals were analytical grade and used without further purification if not indicated otherwise. Dulbecco’s modified eagle’s medium (DMEM) was purchased from GE Healthcare Life Sciences HyClone Laboratories (HyClone, USA). Fetal bovine serum (FBS) was purchased from VivaCell (Shanghai, China), and Trypsin EDTA Solution A was purchased from Biological Industries (BI, Israel). Cell Counting kit-8 (CCK-8) was purchased from Fdbio science CO., Ltd. (Hangzhou, China) and used as received. Dialysis tubes were purchased from Shanghai Lvniao Technology Group. Clear polystyrene tissue culture treated 6-well and 96-well plates were purchased from JET BIOFIL CO., Ltd. (Guangzhou, China). The deionized water was used in the experiment, which was purified from Milli-Q (Millipore, 18.2 MΩ cm^−1^).

### Cells and animals

The human squamous carcinoma cells WSU-HN6, Murine macrophage-like cells RAW 264.7, human umbilical vein endothelial cells (HUVEC) and human cervical cancer cells (HeLa) were cultured as previously described [45, 46]. All specific pathogen free (SPF) mice were purchased from the Hunan SJA Laboratory Animal CO., LTD (Hunan, China) and housed under pathogen-free environment. All animal protocols were reviewed and approved by the Ethics Committee of the College of Stomatology, Chongqing Medical University (Approval No*.* 2021025). For all the experiments on animals in this research, mice were randomly (random number table method) allocated into each group. Grouping was done according to the experimental designs.

### Cell membrane fragments fusion study

Before fusion, WSU-HN6 and RBC membrane fragments were prepared following the previously reported methods (See supplementary methods in Additional file 1). FÖrster resonance energy transfer (FRET) was employed to evaluate the erythrocyte and cancer cell membrane fusion process. Briefly, WSU-HN6 membranes was stained with DiI (detected at an excitation of 549 nm and emission of 565 nm) and DiO (Ex. 484 nm and Em. 501 nm). According to the theory that the weight of cell membrane was the double of membrane protein weight, erythrocyte membrane was added to WSU-HN6 membrane at erythrocyte membrane to WSU-HN6 membrane protein weight rations of 0:1, 1:1, 3:1,and 5:1, respectively [34]. Each group was sonicated for 1 min in an ice bath. After sonication, Samples were extruded through 1.0 × 10^3^ nm, 400 nm, and 200 nm polycarbonate porous membrane to promote fusion of the two kinds of cell membrane. Finally, RBC-H hybrid membrane vesicles were obtained by centrifugation at 14,000×*g* for 0.5 h at 4 ℃ and resuspended in DEPC solution. Fluorescence spectrum for each group was recorded from 500 to 750 nm with an excitation wavelength at 484 nm. Fluorescence recovery of the donor (DiI) was used to monitor the changes in the fusion process.

Then, CLSM was employed to evaluate membrane colocalization. Briefly, DiI and DiO were first dissolved in dimethyl sulfoxide (DMSO). Then, 10 µL of DiO solution was added to 100 µL of the WSU-HN6 membrane solution. The mixed sample was stirred for 2 h away from light in an ice bath. Similarly, 10 µL of the DiI solution was added to 100 µL of RBC membrane solution. The sample was also stirred for 2 h away from light in an ice bath. After thorough mixture, dye labeled cell membrane solutions were centrifuged at 14,000×*g* for 0.5 h at 4℃ and washed four times using PBS to remove free DiO and DiI dye. To fuse the two types of cell membranes, DiO-labeled WSU-HN6 membrane and DiI-labeled erythrocyte membrane were mixed in an ice bathe and sonicated for 2 min. After sonication, the mixed solution was extruded though a 1 μm, 400 nm, 200 nm polycarbonate porous membrane to prepare hybrid membrane fusion. Finally, the RBC-H hybrid membrane vesicles were obtained by centrifuged at 14,000×*g* for 0.5 h at 4 ℃ and resuspended in DEPC water. The RBC-M hybrid membrane (10 µL) was placed on a glass slide, naturally evaporated, and subjected to CLSM. DiI was excited using a 548 nm laser and the red mission collected at 565 nm. A physical mixture of DiO-ladeled WSU-HN6 membrane and DiI-labeled erythrocyte membrane without extrusion was employed as the control. To further validate the successfully fusion of both cell membranes, the immunogold staining assay was performed (see Methods in Additional file 1).

### Preparation and characterization of biomimetic NPs

Synthesis of polyester H40-poly(ethylene glycol) (H40-PEG) and H40-PEG NPs were performed as previously described [47]. H40-PEG loading TPZ/IR780 nanoparticles were prepared using the oil-in-water emulsion solvent diffusion method as follows: H40-PEG (25 mg) and IR780 (2.5 mg) were dissolved in 5 mL of chloroform while TPZ was dissolved in 5 mL of deionized water [48]. Both solutions were mixed and ultrasonicated. After ultrasonication for 1 h in an ice bath, H40-TPZ/IR780 NPs were successfully synthesized. Brown precipitates were collected by centrifugation. Next, NPs were washed three times using deionized water for further analysis. The whole reaction was performed away from light.

RBC cell and WSU-HN6 cell membranes were mixed at a membrane weight ratio of 1:1. The mixture was sonicated at power level of 60 W for 5 min with a sequence of 1 min of sonication and 2 min of break in an ice bath. The RBC-H fused hybrid membrane was coated onto H40-PEG NPs or H40-TPZ/IR780 NPs through the extrusion method. The sonicated RBC-H hybrid membrane solution was added to H40-PEG NPs or H40-TPZ/IR780 NPs solution, and the mixture was successively extruded through 1 μm, 400 nm and 200 nm polycarbonate porous membrane to form hybrid membrane-coated NPs. For Asp8[H40-PEG@(RBC-H)] NPs preparation, DSPE-PEG2000-Asp8 (10%, molar ratio) was incubated with H40-PEG@(RBC-H) NPs solution with stirring at 37 ℃ for 2 h. Finally, biomimetic NPs were obtained by centrifugation (10,000 rpm, 5 min, 4 ℃), washed three times with PBS, and resuspended in a buffer solution for further characterization.

### Characterization of membrane protein

To evaluate membrane protein profiles of RBCs, WSU-HN6, H40-PEG@(RBC-H) NPs and Asp8[H40-PEG@(RBC-H)] NPs, sodium dodecyl sulfate-polyacrylamide gel electrophoresis (SDS-PAGE) assay was performed. Briefly, the membrane and cytosol protein extraction kit (Beyotime, China) was used to separate the surface proteins from RBC cells, WSU-HN6 cells, and hybrid membrane (RBC-H). Membrane protein samples were mixed with SDS-PAGE sample loading buffer (5×, Beyotime, China) and heated for 10 min at 100 ℃. Then, protein samples were loaded on 8% SDS-PAGE gels (Beyotime, China). After running at 80 V for 30 min, voltage was increased to 120 V and samples kept running for about 90 min. Coomassie brilliant blue was used to stain the resulting gels for 2 h. Stained gels were washed for 12 h, and the decolorizing solution was changed thrice during washing. Treated gels were captured by ChemiDOC^TM^XRS+System (Bio-Rad, USA).

To further characterize specific protein expression of cell membranes, resultant gels were transferred to polyvinylidene difluoride (PVDF) membranes (0.45 μm,Servicebio, China) for Western blot analysis. After blocking with 5% skimmed milk, PVDF membranes were probed with anti-CD47 (66304-1-Ig, Proteintech, Wuhan, China) and anti-CD44 (60224-1-Ig, Proteintech, Wuhan, China) overnight at 4 ℃. The PVDF membranes was incubated with horseradish peroxidase conjugated affinipure goat anti-mouse IgG (H+L) (SA00001-1, Proteintech, Wuhan, China) secondary antibody at room temperature for 1.5 h and washed three times (5 min time^−1^). Finally, an enhanced chemiluminescence detection kit (Beyotime, China) was used to visualize PVDF membranes. Signals of both special proteins were recorded on the ChemiDOC^TM^XRS+System (Bio-Rad, USA).

### Hydroxyapatite binding assay

To investigate bone-targeted abilities of the biomimetic NPs, hydroxyapatite (HA) binding assay was performed in vitro as previously reported [49]. HA beads were suspended in PBS at 20 mg mL^−1^. Then, 600 µL of different DiI-loaded nanoparticles (DiI as the fluorescent probe with a concentration of 10 μM for each sample) were mixed with 300 µL of HA suspension or 300 µL of the PBS as a control, followed by gentle shaking at 100 rpm for 1 h at 37 ℃. After centrifugation at 10,000×*g* for 5 min, the HA precipitate was separated from unbound nanoparticles in the supernatant. The DiI fluorescence of supernatant was quantified through a multi-functional microporous plate reader (SpectraMAX iD5, USA) at a wavelength of 549 nm. The degree of HA binding was calculated according to the following:

1 $${\text{HA binding rate}} (\%) = (A-B)/A$$

where A is the fluorescence intensity of DiI in the control group and B is the fluorescence intensity of DiI in the experimental groups.

### Specific targeting WSU-HN6 cell line in vitro

HeLa cells, HUVEC cells and WSU-HN6 cells were seeded into 6-well plates with cell climbing slice at a density of 5.0 × 10^4^ cells well^−1^ in 1.5 mL of complete DMEM, respectively. After incubation for 12 h, medium was replaced with fresh medium containing Asp8[H40-PEG@(RBC-H) nanoparticles and DiI-dyed RBC-H (with H40-PEG concentration at 100 µg mL^−1^). After 4 h, cells in all groups were washed twice using cold PBS and fixed using 4% paraformaldehyde (PFM) for 20 min at room temperature. Cold PBS was used to wash cell climbing slices for a total of three times. Nuclei for all cell lines were dyed with DAPI for 5 min and rinsed three times using cold PBS. Finally, all samples were detected with CLSM and flow cytometer (CytoFLEX, Beckman Coulter). Moreover, to further investigate the homotypic targeting effect of this biomimetic NPs with co-incubated WSU-HN6/ HeLa cells, the WSU-HN6 cells were labeled firstly by DiO. Then, both HeLa cells and DiO-labeled WSU-HN6 cells were seeded into a climbing slice at the density 2.5 × 10^4^ cells of each cell line in 1.5 mL of complete DMEM. And the remaining steps were kept the same as mentioned above. Finally, all samples were detected with CLSM.

### Cellular uptake of biomimetic NPs by macrophages

To evaluate macrophage uptake of different nanoparticles, CLSM and flow cytometry were used to determine immune escape abilities of resulting biomimetic NPs in vitro. Briefly, RAW 264.7 macrophage cells were seeded at a density of 1.0 × 10^5^ cells well^−1^ into 6-well plates containing cell climbing slices and 1.5 mL of complete DMEM. Incubation was done at 37 ℃ for 12 h. Cells were incubated with FITC loaded H40-PEG NPs and FITC loaded Asp8[H40-PEG@(RBC-H)] NPs (FITC concentration at 10 µg mL^−1^) for 4 h at 37 ℃. Cells on the climbing slices were washed twice using cold PBS and fixed with 4% PFM for 20 min at room temperature. Then, the cells were rinsed three times using cold PBS. After cell nuclei had been stained with DAPI for 5 min, they were rinsed three times using PBS. Finally, slices were mounted and observed by CLSM. To quantify cellular uptake of the nanoparticles by RAW 264.7 cells, cells were digested with pancreatin after incubation for 4 h. Then, cells were obtained and centrifuged (3.0 × 10^3^ rpm, 3 min, 4 ℃). Finally, harvested cells were suspended in cold PBS, and subjected to flow cytometry (CytoFLEX, Beckman Coulter) for analysis of fluorescence intensities of all groups. Furthermore, to assess the stability of Asp8[H40-PEG@(RBC-H)] NPs, the changes in nanoparticle size were observed through DLS. Briefly, Asp8[H40-PEG@(RBC-H)] NPs was incubated with PBS, and the size changes in size over 72 h were measured. The H40-PEG NPs were used as a control.

### Cellular uptake Asp8[H40-PEG@(RBC-H) NPs

The intracellular uptake assay was performed through CLSM and flow cytometry. As a near-infrared light sensitive dye, IR780 was used as the model drug in the intracellular uptake experiment due to red fluorescence and hydrophobic molecule characteristics.

### CLSM

To visualize the endocytosis process of biomimetic nanoparticles by WSU-HN6 cells, CLSM was used for imaging at preset time points. Briefly, WSU-HN6 cells were seeded in 6-well plates with cell climbing slices at 1.0 × 10^5^ cells well^−1^ in 1.5 mL of complete DMEM and incubated for 12 h. Then, the complete medium was removed, and cells were incubated with Asp8[H40-IR780@(RBC-H)] nanoparticles (1 mL of DMEM medium) at a final IR780 concentration of 10 µg mL^−1^. Cells were incubated for predetermined intervals at 37 ℃. Subsequently, they were washed twice using cold PBS and fixed in 4% PFM for 20 min at room temperature. Slices were rinsed three times using cold PBS. Afterwards, the nuclei of WSU-HN6 cells were stained with DAPI for 5 min. Slices were rinsed three times using PBS. Finally, slices were mounted and observed by CLSM.

### Flow cytometry

WSU-HN6 cells were seeded into 6-well plates at 1.0 × 10^5^ cells well^−1^ in 1.5 mL of complete DMEM and incubated for 12 h. Then, biomimetic NPs were dissolved in DMEM culture medium at a final IR780 concentration of 10 µg mL^−1^. They were added into different wells and incubated for 5, 15, 30, 60, and 240 min, respectively. Thereafter, DMEM was removed, and cells were washed twice using cold PBS and treated with trypsin (200 µL). Subsequently, 2 mL of PBS was added to each well and the solutions were centrifuged at 4 ℃ for 5 min (3.0 × 10^3^ rpm). After removal of the supernatants, 500 µL of cold PBS was used to resuspend the cells. Data for 1.0 × 10^4^ gate events were collected and analyzed by flow cytometer (CytoFLEX, Beckman Coulter) and FLOWJO 10 software.

### TEM and DLS assay

For optimal anticancer efficacy, photosensitizer IR780 and hypoxia-activated chemotherapy drug TPZ were selected model drugs in this study. To characterize Asp8[H40-TPZ/IR780@(RBC-H) nanoparticles, the size and ζ-potential of the nanoparticles were measured by Malvern Zetasizer Nano S in distilled deionized water at room temperature. Then, the size and morphology of the hybrid membrane-camouflaged nanoparticles were directly captured by TEM (Talos F200s instrument Thermoscientific, Czech).

The UV-vis absorption spectra were utilized to ensure successful encapsulation of IR780 and TPZ in Asp8[H40-PEG@(RBC-H) NPs. Standard curves for IR780 and TPZ were determined by UV-vis spectrophotometry. Drug loading and encapsulation efficiencies of both IR780 and TPZ were respectively calculated through the corresponding standard curve. Drug loading and encapsulation efficiencies were determined as:

2 $$\text{Drug loading efficiency (DLE)} =\, \text{(Weight of the drug in resulting nanoparticles}/\text{Weight of the nanoparticles)} \times 100 \%.$$

3 $$\text{Drug encapsulation efficiency} = \text{(Weight of the drug in nanoparticles}{/} \text{Weight of the feeding drug)} \times 100\%.$$

Moreover, TPZ and IR780 release rates from Asp8[H40-TPZ/IR780@(RBC-H) NPs were determined using dialysis tubes containing PBS buffer (0.01 M, pH=7.4) and acetate buffer (0.01 M, pH = 5.0), respectively. Briefly, Asp8[H40-TPZ/IR780@(RBC-H) NPs (2.0 mg mL^−1^, 2.0 mL) were placed in dialysis tubes (MWCO=3500 Da) and soaked in 50 mL of both aforementioned buffer solutions, respectively. Then, they were placed in a 37 ℃ constant temperature shaker at 100 rpm. Dialysate (2.0 mL) was obtained at designated time points. Moreover, 2.0 mL of the fresh supplement buffer was added to replace the sampled buffer. TPZ or IR780 concentrations of samples were measured to determine release rates of corresponding drug contents through a UV-vis spectrophotometer. To investigate whether near infrared light-triggered the drug release, both buffer solutions were irradiated under 808 nm laser for 5 min (1.0 W cm^−2^) and 2.0 mL of solution was obtained at designated time points. The remaining process was performed as earlier described.

### Hemolysis assay

To evaluate the safety of biomimetic NPs, a hemolysis experiment was performed. Briefly, fresh BALB/c blood was obtained in heparin sodium-containing tubes. RBCs were separated and collected by centrifugation at 3.0 × 10^3^ rpm for 10 min at 4 ℃ and washed several times using ice-cold PBS. The process was repeated more than three times to make sure the supernatant was colorless. Then, the RBCs were diluted using ice-cold PBS to a concentration of 2 % (v/v). Biomimetic nanoparticles and control polymers (Triton X-100 and Dextran) were prepared at serial concentrations (0.02, 0.05, 0.1, 0.2, 0.5, 1.0, and 2.0 mg mL^−1^) using PBS. Then, 0.5 mL of the biomimetic NPs and control polymers were added to 0.5 mL of the 2 % (v/v) RBC solution with gentle shaking at 37 ℃. After 1 h of incubation, samples were centrifuged at 1.0 × 10^4^ rpm for 10 min at 4 ℃, Supernatants (200 µL) were collected and transferred to 96-well plate. About 1% of triton X-100 solution (v/v, 100% lysis) was used as positive control while Dextran was utilized as the negative control. Hemoglobin absorbance was measured using a multi-functional microporous plate reader at a wavelength of 545 nm. Hemolysis degree was assessed as a mean of three independent experiments using Eq. (4): 

4 $$\text{The hemolysis percent} = (OD_{\text{sample}}-OD_{\text{negative}})/(OD_{\text{positve}}-OD_{\text{negative}}) \times100 \%.$$

### ROS/hypoxia detection in vitro

To assess the production of intracellular ROS/hypoxia, a ROS-ID® Hypoxia/Oxidative Stress Detection Kit (ENZO, USA) was used, according to the manufacturer’s instructions [50]. In briefly, WSU-HN6 cells were seeded into a laser confocal cell culture dish at a density of 5.0 × 10^4^ per well in 1.0 mL of complete DMEM. After incubation for 12 h, cells were treated as follows: (i) The medium was replaced with fresh completed DMEM (1.0 mL) containing PBS, which was used as negative control group; (ii) Medium was replaced with fresh completed DMEM (1.0 mL) containing Asp8[H40-TPZ/IR780@(RBC-H)] NPs (50 µg mL^− 1^ of IR780, according to IR780) for 4 h without or (iii) with a laser irradiation (808 nm, 1.0 W cm^− 2^, 5 min); (iv) The medium was replaced with fresh completed DMEM (1.0 mL) containing an ROS inducer pyocyanin (Pyo), for 30 min or (v) a hypoxia inducer, deferoxamine (DFO), for 3.5 h, respectively. Then, the cells were incubated with both hypoxia probes and ROS probes for 10 min and observed utilizing CLSM. The ROS green fluorescence and hypoxic red fluorescence signals were detected by CLSM with excitation wavelengths of 488 nm and 561 nm, respectively.

### Cytotoxicity and antitumor efficacies in vitro

HUVEC cells and WSU-HN6 cells were respectively cultured in DMEM supplemented with 10 % FBS, 100 U/mL streptomycin and 100 U/mL penicillin at 37 ℃ in a humidified incubator containing 5% CO_2_ (Thermo Scientific, USA). Relative cytotoxicity of Asp8[H40-PEG@(RBC-H)] biomimetic NPs against both cells were assessed through CCK-8 assay. Briefly, HUVEC cells and WSU-HN6 cells were seeded in 96-well plates at a density of 4.0 × 10^3^ cells/well in 200 µL of complete medium for 24 h at 37 ℃. Then, the culture medium was removed and replaced with 200 µL of the medium containing serial of concentrations of the biomimetic nanoparticles. The final concentration was in the range of 5-40 µg mL^−1^. At least three wells were prepared for each dose of nanoparticles. These cells were incubated for another 48 h. The CCK-8 solution was added into each well, and the 96-well plates were incubated for 1 h at 37 ℃. Absorbance value for each well was measured using a multi-functional microporous plate reader (SpectraMAX iD5, USA) at a wavelength of 450 nm.

To assess the anticancer effects of biomimetic NPs in vitro, WSU-HN6 cells were seeded into 96-well plates at a density of 4.0 × 10^3^ cells /well in 200 µL of complete medium. Subsequently, cells were allocated into two groups, including normoxia-therapy group and the hypoxia-therapy group. Cells in the hypoxia-therapy group were transferred from normoxic condition (20% O_2_) to hypoxic chamber (1% O_2_). Incubation was performed for an additional 12 h. Then, 200 µL of fresh medium containing different concentrations of biomimetic NPs were added to replace the spent medium (n = 3 per group). After incubation in hypoxia or normoxia for 6 h away from light, cells were washed twice using cold PBS. Subsequently, the culture medium was replaced with PBS, and cells were either irradiated with the laser (808 nm, 1.0 W cm^−2^, 5 min) or not. After incubation for 24 h in normoxic conditions, the ability of WSU-HN6 cells was determined by performing the CCK-8 assay. Procedures for the CCK-8 assay as described above.

### Calcein-AM/PI double staining assay

To assess the viability of WSU-HN6 cells in different nanoparticles, calcein-AM/PI double staining was performed and observed by fluorescence microscope (EVOS FL Auto, USA). Briefly, WSU-HN6 cells (5.0 × 10^4^ cells well^−1^) were seeded in 12-well plates in 1.5 mL of complete medium. After culturing 12 h, different concentrations nanoparticles and PBS were added to the wells for 12 h. And cells in all groups were irradiated with 808 nm laser for 5 min, or not. Finally, calcein-AM/PI double staining was carried out, and fluorescence images of the live and dead cells were imaged by fluorescence microscope (EVOS FL Auto, USA).

### Cell apoptosis in vitro

To determine the ability of various nanoparticles with and without laser, an Annexin V-FITC/PI apoptosis detection kit and flow cytometry were used in this study [51]. Briefly, WSU-HN6 cells were seeded into 6-well plates at a density of 5.0 × 10^4^ per well in 1.0 mL of complete DMEM. After incubated for 24 h at 37 ℃, cells were grouped and treated differently, including with PBS, H40-PEG loading IR780 NPs, Asp8[H40-IR780@(RBC-H)] NPs, and Asp8[H40-TPZ/IR780@(RBC-H)] NPs (10 µg mL^−1^ of IR780). The PBS group was set as the control. After incubation 4 h, suspensions of all groups were replaced with fresh complete DMEM. Cells in laser groups were irradiated with 808 nm laser for 5 min (1.0 W cm^−2^). Then, cells in all groups were cultured in fresh complete DMEM at 37℃ for another 4 h and treated with the apoptosis Analysis Kit. Finally, apoptotic cells were analyzed by flow cytometry (CytoFLEX, Beckman Coulter) and corresponding software.

### Infrared imaging in vitro

To evaluate photothermal characteristics of biomimetic NPs in vitro, a thermal imaging camera (FOTRIC 365 C, Shanghai, China) was used to obtain thermal images under irradiation using an 808 nm laser for 5 min with serial power densities (0.5 W cm^−2^, 0.6 W cm^−2^, 1.0 W cm^−2^, 1.2 W cm^−2^, 1.5 W cm^−2^). Biomimetic NPs with different IR780 concentrations (0, 50 µg mL^−1^, 100 µg mL^−1^) in 12-well plate were further irradiated with the 808 nm laser (1.0 W cm^−2^, 5 min).

### Infrared imaging in vivo

To evaluate photothermal effects of the biomimetic NPs, an infrared thermal camera was used to determine effective therapy in vivo [52]. Briefly, tumor-bearing mice were intravenously injected with Asp8[H40-TPZ/IR780@(RBC-H)] NPs (1.6 mg Kg^−1^ of IR780 dose) or normal saline. Then, 8 h after injection, mice were irradiated by laser for 5 min (808nm, 1.0 W cm^−2^). The Fotric AnalyzIR software was utilized to analyze the thermographs.

### Biodistribution in vivo

To assess nanoparticle biodistribution in bone invasion models, DiD, a lipophilic near-infrared fluorescent dye, was used to mark different nanoparticles [53]. Tumor-bearing nude mice were intravenously administered with serial DiD-loaded nanoparticles or normal saline through the tail vein at a prescribed DiD dose (50 µg mL^−1^). Then, 8 h after injection, mice were sacrificed, and the main organs, including the heart, liver, spleen, lung, kidney, the lesion and healthy mandibula were carefully dissected, rinsed using normal saline, and stored away from light for further analysis. DiD fluorescence visualization of each organ (Ex: 655 nm, Em: 714 nm) was performed using an imaging system (VISQUE In Vivo Smart).

### Therapeutic evaluation of Asp8[H40-TPZ/IR780@(RBC-H) in vivo

To evaluate anticancer efficacy of various TPZ/IR780-loaded NPs in a bone invasion model, mouse model whose right mandibular had been invaded were selected as experimental animals. Model were developed as previously reported with minor alterations [54]. Briefly, 2.0 × 10^6^ WSU-HN6 cells were injected into the right masseter region of female BALB/c nude mice (4-6 weeks old). After 1 week, tumor formation rates were determined to be 100%. Then, mice were randomly assigned into 7 groups (five mice per group), including, (i) Normal saline, (ii) Normal saline+laser, (iii) TPZ+laser, (iv) Asp8[H40-PEG@(RBC-H)] NPs+laser, (v) H40-PEG loading IR780 NPs+laser, (vi) H40-TPZ/IR780@(RBC-H) NPs+laser, and (vii) Asp8[H40-TPZ/IR780@(RBC-H)] NPs+laser. Mice in all groups were administrated with 100 µL PBS or different formulations at a TPZ dose of 1.5 mg Kg^−1^ and a IR780 dose of 1.6 mg kg^−1^ through the tail vein. Mice in the PBS group were used as negative controls. The first day of administration was designated as day 0. Tumor volumes and body weights of mice were measured every day. After 24 h, mice in the laser group were illuminated with 808 nm wavelength laser irradiation (1.0 W cm^−2^, 5 min) once per day for 2 consecutive days. Tumor volumes (V) were determined using Eq. (5): 

5 $$V = {Width^{2}} \times Length/2.$$

According to ethical reasons, mice with tumor sizes exceeding 150 mm in any dimension were sacrificed. Otherwise, mice were euthanized on day 16, tumors were dissected, weighted, photographed, and fixed in 4% paraformaldehyde for further analysis. Hematoxylin and eosin (H&E) staining and fluorescein (FITC) terminal deoxynucleotidyltransferase-mediated UTP end labeling assay (TUNEL, Servicebio, China) were used to evaluate the changes of cancer tissue and to detect apoptosis in tumor slices.

### Micro-computed tomography reconstruction of right mandibula

To assess the degrees of mandibula destruction by cancer, micro-computed tomography (micro-CT) scanning was used to scan the right mandibula of all mice. Mice were euthanized on day 16 after which tumor-bearing mandibula were carefully dissected and placed into 4 % PFM solution. Afterwards, each specimen was scanned using a micro-CT scanner (Scanco VivaCT40, Switzerland). Three-dimensional models were reconstructed and analyzed using a Scanco VivaCT40 micro-CT software. And relative bone loss volume was calculated using Eq. (6): 

6 $$\text{The relative bone loss volume percent} = \text{(Blank group}-\text{experimental group)}{/} \text{Blank group} \times 100\%.$$

### Systemic toxicity evaluation

To assess the biosafety of biomimetic NPs during therapy, besides measuring body weights of mice every day, changes in blood and major organs are also important indicators [55]. Mice were sacrificed after 16 days of treatment. Blood samples were obtained from the orbital venous before sacrifice and analyzed at the stomatological hospital of Chongqing medical university. Moreover, major organs including the heart, liver, spleen, lung, and kidney were carefully dissected and fixed with 4% PFM for further H&E staining and analysis.

### Statistical methods

Unless otherwise stated, statistics were determined via a one-way ANOVA with Tukey multiple comparisons test using GraphPad Prism Version 8.0.1 software. All reported data show means and standard error unless otherwise noted. *P < 0.05, **P < 0.01, and ***P < 0.001 were determined statistically significant with n ≥ 3 for all experiments. And ns indicates no significant difference (*P* > 0.05).

## Supplementary Information

**Additional file 1.** Materials and reagent, characterization, preparation of erythrocyte and cancer cell membrane fragments, immunogold staining assay, and TPZ/IR780 loading and release in vitro. **Figure S1.** Confocal laser florescent microscopy of images of RBC membrane, WSU-HN6 membrane, and fused RBC-H hybrid membrane vesicles. **Figure S2.** Quantitative analysis of the FITC fluorescence intensity. **Figure S3.** The average size of Asp8[H40-PEG@(RBC-H)] NPs was monitored by DLS before and after lyophilization. **Figure S4.** Release profiles of TPZ from the Asp8[H40-TPZ/IR780@(RBC-H)] NPs in different pH (5.0 and 7.4) buffer with or without 808 nm laser irradiation (1.0 W cm^−2^, 5 min). **Figure S5.** The cell viability of Asp8[H40-PEG@(RBC-H)] NPs against HUVEC cells after cultured for 72 h with series of concentrations. **Figure S6.** The cell viability of Asp8[H40-PEG@(RBC-H)] NPs against WSU-HN6 cells after cultured for 72 h with series of concentrations. **Figure S7.** The increasing temperature photographs of normal saline, 10% FBS, PBS, and Asp8[H40-TPZ/IR780@(RBC-H)] NPs (the concentration of IR780 = 100 µg mL^−1^ ) upon 808 nm laser irradiation (1.0 W cm^−2^, 5 min) in vitro.

## Data Availability

All data generated or analyzed during this study are included in this published article and its Additional file 1.

## Abbreviations

TPZ: Tirapazamine
NPs: Nanoparticles
H: WSU-HN6 cell
RBC: Red blood cell
Asp8: Oligopeptide of eight aspartate
BPs: Bisphosphonates
BRONJ: Bisphosphonate-related osteonecrosis of the jaw
FRET: FÖrst resonance energy transfer
TEM: Transmission electron microscopy
H&E: Hematoxylin and eosin
DiD: 1,1′-Dioctadecyl-3,3,3′,3′-tetramethylindodicarbocyanine,4-cholrobenzenesulfonate
PLGA: Poly(lactic-co-glycolic acid)
EPR: Enhanced permeability and retention
DiI: 1,1′-dioctadecyl-3,3,3′-tetramethylindocarbocyanine prechlorate
OSCC: Oral squamous cell carcinoma
DiO: 3,3′-dioctadecyloxacarbocyanine perchlorate
CLSM: Confocal laser scanning microscopy
PBS: Phosphate buffer saline
ROS: Reactive oxygen species
RES: Reticuloendothelial system
PTT: Photothermal treatment
HA: Hydroxyapatite
