# Correction to: Bone‑targeted erythrocyte‑cancer hybrid membrane‑camouflaged nanoparticles for enhancing photothermal and hypoxia‑activated chemotherapy of bone invasion by OSCC

**DOI:** 10.1186/s12951-021-01185-9

**Published:** 2022-01-03

**Authors:** Hongying Chen, Jiang Deng, Xintong Yao, Yungang He, Hanyue Li, Zhixiang Jian, Yi Tang, Xiaoqing Zhang, Jingqing Zhang, Hongwei Dai

**Affiliations:** 1 College of Stomatology, Chongqing Medical University, Chongqing, 401147 China; 2 Chongqing Key Laboratory of Oral Diseases and Biomedical Sciences, Chongqing, 401147 China; 3 Chongqing Municipal Key Laboratory of Oral Biomedical Engineering of Higher Education, Chongqing, 401147 China; 4 Department of Pharmacology, School of Pharmacy, Chongqing Medical University, Chongqing, 400016 China; 5 Key Laboratory of Biochemistry and Molecular Pharmacology of Chongqing, Chongqing Medical University, Chongqing, 400016 China; 6 Chongqing Research Center for Pharmaceutical Engineering, Chongqing Medical University, Chongqing, 400016 China

## Correction to: J Nanobiotechnol (2021) 19:342 10.1186/s12951-021-01088-9

Following publication of the original article [1], the authors identified an error in Fig. 8g. The correct figure is given in this erratum.

Original data:

Corrected Fig. 8:

**Fig. 8:**
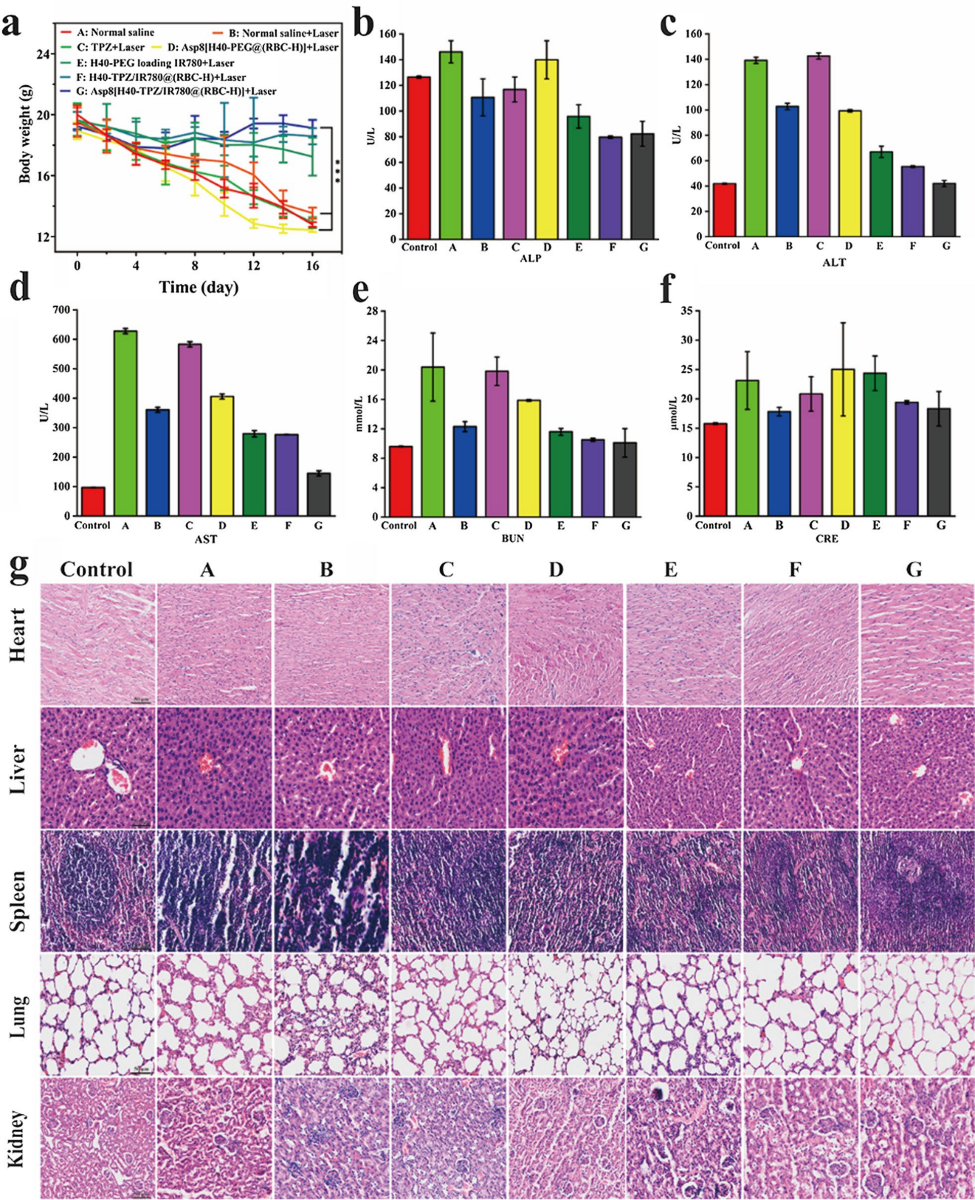
Preliminary biosafety assay of Asp8[H40-TPZ/IR780@(RBC-H)] NPs. **a** The body weight change curves of nude mice bearing WSU-HN6 tumor after various treatment (n = 5, mean ± SD). **b**–**d** Main blood biochemical parameters of liver function including ALP, ALT, and AST. **e**, **f** Main biochemical parameters of kidney function including BUN and CRE. **g** H&E staining slices of major organs including heart, liver, spleen, lung, and kidney from each group. The mice without any therapy were as a blank group (scale bar = 50 μm). (A: Normal saline, B: Normal saline + Laser, C: TPZ + Laser, D: Asp8[H40-PEG@(RBC-H)] + Laser, E: H40-PEG loading IR780 + Laser, F: H40-TPZ/IR780@(RBC-H) + Laser, and G: Asp8[H40-TPZ/IR780@(RBC-H)] + Laser). ***p < 0.001

Furthermore, an error was identified in the Methods on Line 5 on page 23. The correct version is given in this erratum.

These cells were incubated for another 72 h.

“The authors apologise for this error”.
