# Supplementary material for: Bone-targeted erythrocyte-cancer hybrid membrane-camouflaged nanoparticles for enhancing photothermal and hypoxia-activated chemotherapy of bone invasion by OSCC
Source: J Nanobiotechnology. 2021 Oct 26;19:342. doi: 10.1186/s12951-021-01088-9 (PMC8549398; doi:10.1186/s12951-021-01088-9)
Supplement: Supplementary file 1 — Additional file 1. Materials and reagent, characterization, preparation of erythrocyte and cancer cell membrane fragments, immunogold staining assay, and TPZ/IR780 loading and release in vitro. Figure S1. Confocal laser florescent microscopy of images of RBC membrane, WSU-HN6 membrane, and fused RBC-H hybrid membrane vesicles. Figure S2. Quantitative analysis of the FITC fluorescence intensity. Figure S3. The average size of Asp8[H40-PEG@(RBC-H)] NPs was monitored by DLS before and after lyophilization. Figure S4. Release profiles of TPZ from the Asp8[H40-TPZ/IR780@(RBC-H)] NPs in different pH (5.0 and 7.4) buffer with or without 808 nm laser irradiation (1.0 W cm−2, 5 min). Figure S5. The cell viability of Asp8[H40-PEG@(RBC-H)] NPs against HUVEC cells after cultured for 72 h with series of concentrations. Figure S6. The cell viability of Asp8[H40-PEG@(RBC-H)] NPs against WSU-HN6 cells after cultured for 72 h with series of concentrations. Figure S7. The increasing temperature photographs of normal saline, 10% FBS, PBS, and Asp8[H40-TPZ/IR780@(RBC-H)] NPs (the concentration of IR780 = 100 µg mL−1 ) upon 808 nm laser irradiation (1.0 W cm−2, 5 min) in vitro. [file 12951_2021_1088_MOESM1_ESM.docx]

**Bone-targeted erythrocyte-cancer hybrid membrane-camouflaged nanoparticles for enhancing photothermal and hypoxia-activated chemotherapy of bone invasion by OSCC**

Hongying Chen^1,2,3^, Jiang Deng^1,2,3^, Xintong Yao^4,5^, Yungang He^1,2,3^, Hanyue Li^1,2,3^, Zhixiang Jian^1,2,3^, Yi Tang^1,2,3^, Xiaoqing Zhang^1,2,3^, Jingqing Zhang^6*^, and Hongwei Dai^1,2,3*^

^1^College of Stomatology, Chongqing Medical University, Chongqing, 401147, China.

^2^Chongqing Key Laboratory of Oral Diseases and Biomedical Sciences, Chongqing, 401147, China.

^3^Chongqing Municipal Key Laboratory of Oral Biomedical Engineering of Higher Education, Chongqing, 401147, China.

^4^Department of Pharmacology, School of Pharmacy, Chongqing Medical University, Chongqing, 400016, China.

^5^Key Laboratory of Biochemistry and Molecular Pharmacology of Chongqing, Chongqing Medical University, Chongqing, 400016, China.

^6^Chongqing Research Center for Pharmaceutical Engineering, Chongqing Medical University, Chongqing, 400016, China.

*Correspondence: 13308300303@163.com; dai64@hospital.cqmu.edu.cn

**Supplementary Method**s

**Materials and Reagents:**

Dendritic aliphatic polyester H40 was purchased from Weihai CY Dendrimer Technology Co., Ltd. (Weihai, China) and purified as previously described [1]. Anhydrous dimethyl sulfoxide (DMSO) was purchased from Shanghai Macklin Biochemical Co., Ltd. 4-Dimethylaminopyridine (DMAP), triethylamine (TEA), *N*-(3-dimethyl-aminopropyl)-N-ethylcarbodiimide hydrochoride (EDC) were purchased from Sigma-Aldrich and used as received. Asp8 modified 1,2-distearoyl-sn-glycero-3-phosphoethanolamine-*N*-[amino-(poly-ethylene glycol)-2000] (DSPE-PEG2000-Asp8) was purchased from ChinaPeptides Co., Ltd. (Suzhou, China) and used as received. Carboxylated polyethylene glycol (mPEG2000-COOH) was purchased from MeloPEG Science and Technology Co., Ltd. (Shenzhen, China) and used as received. All other chemicals were purchased from Chongqing Chuandong Chemical (group) CO., Ltd. and used without further purification. All chemicals were analytical grade and used without further purification if not indicated otherwise.

**Characterization:**

DLS and zeta potential analysis were performed on a Malvern Zetasizer Nano S device equipped with a 4.0 mw laser operating at λ=633 nm. All samples were measured with a scattering angle of 173° and at 37 ℃. The morphologies of H40-PEG NPs, and Asp8[H40-TPZ/IR780@(RBC-H)] NPs were measured with a Talos F200s instrument (Thermoscientific, Czech) operated at 200 kV. And the samples were prepared by directly dropping the solution onto carbon-coated copper grids and then airdrying at room temperature overnight before measurement. The UV-vis absorption analysis was recorded with a Model UV1200 UV-vis spectrophotometer (Shanghai, China).All fluorescence photographs were obtained on a confocal laser scanning florescence microscope (LSM 510 META, Leica, Germany). The fluorescence spectra were measured on a QM/MT/RM fluorescence spectrophotometer (Photon Technology International, Inc.) at room temperature.

**Preparation of Erythrocyte Cell MembraneFragments:**

Cell membrane fragments were obtained from RBC cells as previously described with minor modifications [2]. Whole blood was obtained from BALB/c nude mice (six weeks) through orbital sinus puncture and collected in sodium heparin-containing tubes. Then, blood was centrifuged at 3 × 10^3^ rpm for 5 min at 4 ℃（Centrifuge 5810 R, Eppendorf, Germany）to remove the serum. Collected erythrocyte precipitate and washed thrice cold phosphate buffered saline (PBS 0.01M, pH=7.4) to remove residual plasm. For hypotonic lysis of the erythrocyte, dilute 1/4 × PBS (pH=7.4) containing 0.2 mM EDTA were added into the tubes to resuspend the erythrocyte in 4 ℃ for 1 hour. Then, lysis solution was centrifuged at 1.3 × 10^4^ rpm for 5 min at 4 ℃ and the supernatant carefully removed. Erythrocyte membrane fragments were obtained as pink precipitates after three washing-centrifugation cycles in cold PBS. Obtained RBC membranes were stored at -80 ℃ for further use. Concentrations of erythrocyte membrane proteins were quantified using the BCA protein assay kit (Beyotime Biotechnology, Shanghai, China), according to the manufacturer’s instructions.

**Preparation Cancer Cell Membrane Fragments:**

Cancer cell membrane fragments of WSU-HN6 cells were prepared as previously described with minor changes [3]. The membrane protein extraction kit (Beyotime Biotechnology, Shanghai, China) was used to extract membrane fragments. Briefly, WSU-HN6 cells were firstly incubated in cell culture dishes with diameter of 15 cm containing complete DMEM (10% (v/v) FBS, 1% penicillin and streptomycin) at 37 ℃ in a humidified atmosphere containing 5% CO_2_. After 48 h, cells were gently obtained with the help of cell rubber scraper. After being washed twice using cold PBS at 4 ℃, cancer cells were obtained by centrifugation at 600 × *g* for 5 min at 4 ℃. The 1mM PMSF membrane protein extraction reagent A was used to resuspend cancer cells. Then, the suspension was cooled in an ice bath for 30 min after which it was sonicated for 1 min (pulse: on-time 2 s and off-time 3 s) in ice bath (power: 60 W, Fisher Scientific, Waltham, MA, USA). The resultant solution was centrifuged at 700 × *g* for 10 min at 4 ℃ to remove unbroken cells or cell debris. Then, the upper layer was carefully pipetted out and centrifuged at 14000 × *g* for 0.5 h at 4 ℃. The obtained WSU-HN6 cell membrane fragments were stored at -80 ℃ for further analysis. Concentrations of WSU-HN6 cell membrane proteins were quantified through the BCA protein assay kit.

**Immunogold Staining Assay:**

To validate the successfully fusion of both cell membranes, the immunogold staining assay was performed as previously reported [4]. Briefly, RBC-H hybrid membrane vesicles solution was added to 4 % paraformaldehyde solution at 1:1 (V/V) ratio. And drop of the mixed solution was deposited onto the copper grid 300 mesh for 20 min and air-dried at room temperature. The copper mesh was soaked in PBS contained 50 mM glycine for 3 min and washed 2 times (3 min time^-1^). Then, it was transferred to PBS and 50 mM glycine for 3 min, respectively. The procedure was repeated 3 times. Next, 5% bovine serum albumin (BSA, Mengbio, China) was used to block the copper mesh. After blocked for 10 min, the copper mesh was incubated with the primary antibodies against CD47 (bs-238R, Rabbit Anti-CD47 Polyclonal Antibody, Bioss, China, 1:500) and CD44 (60224-1-Ig, Proteintech, Wuhan, China, 1:500) for 30 min, and washed six times using BSA (3 min time^-1^).Then, 10 nm of colloidal gold, which was conjugated to secondary antibodies against rabbit (bs-0295G, Goat Anti-Rabbit IgG, Bioss, China, 1:200) IgG, was used to stain the copper mesh for 30 min. The copper mesh was washed six times using PBS and 0.5% BSA (3 min time^-1^), respectively. Glutaraldehyde (1%, Solarbio life sciences) was used to fix the copper mesh for 2 min, then, the distilled water was used to wash the copper mesh 8 time (2 min time^-1^). When performing the secondary antibody of 5 nm size of colloidal gold (bs-0296G, Goat Anti-mouse IgG, Bioss, China, 1:200), this process was repeated again. Finally, the cooper mesh was negatively stained by 3% phosphotungstic acid for 30 s. the samples were examined by TEM (Talos F200s instrument Thermoscientific, Czech).

**Supplementary Figures**


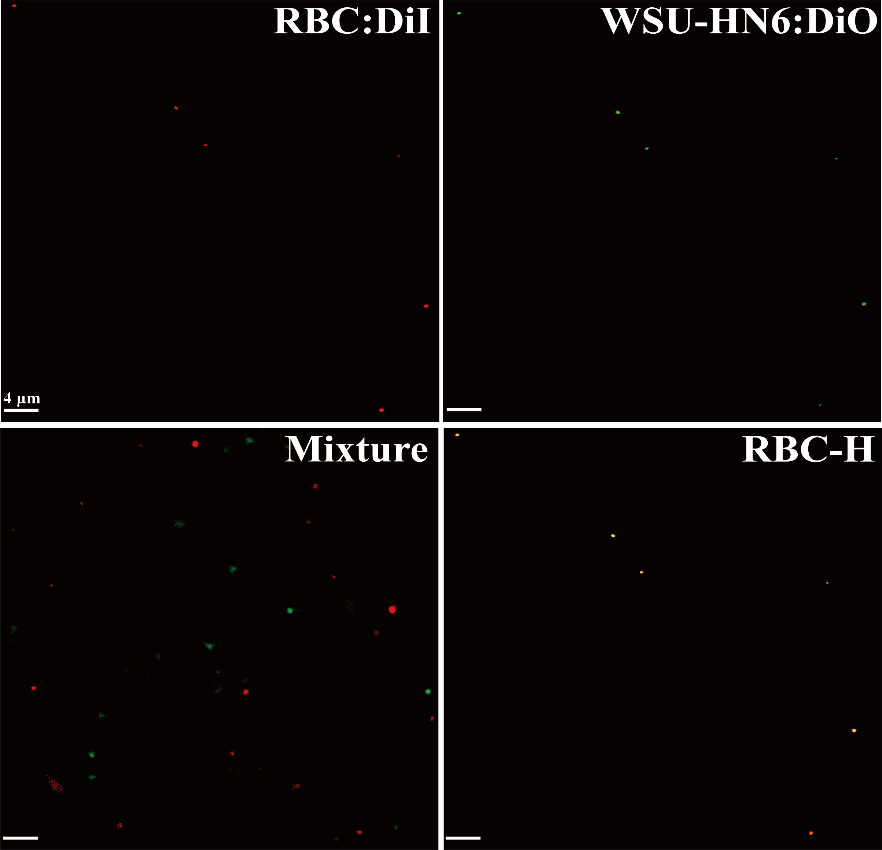


**Figure S1**. Confocal laser florescent microscopy of images of RBC membrane, WSU-HN6 membrane, and fused RBC-H hybrid membrane vesicles. (Red = RBC membrane, Green = WSU-HN6 membrane; Scale bar = 4 μm ).


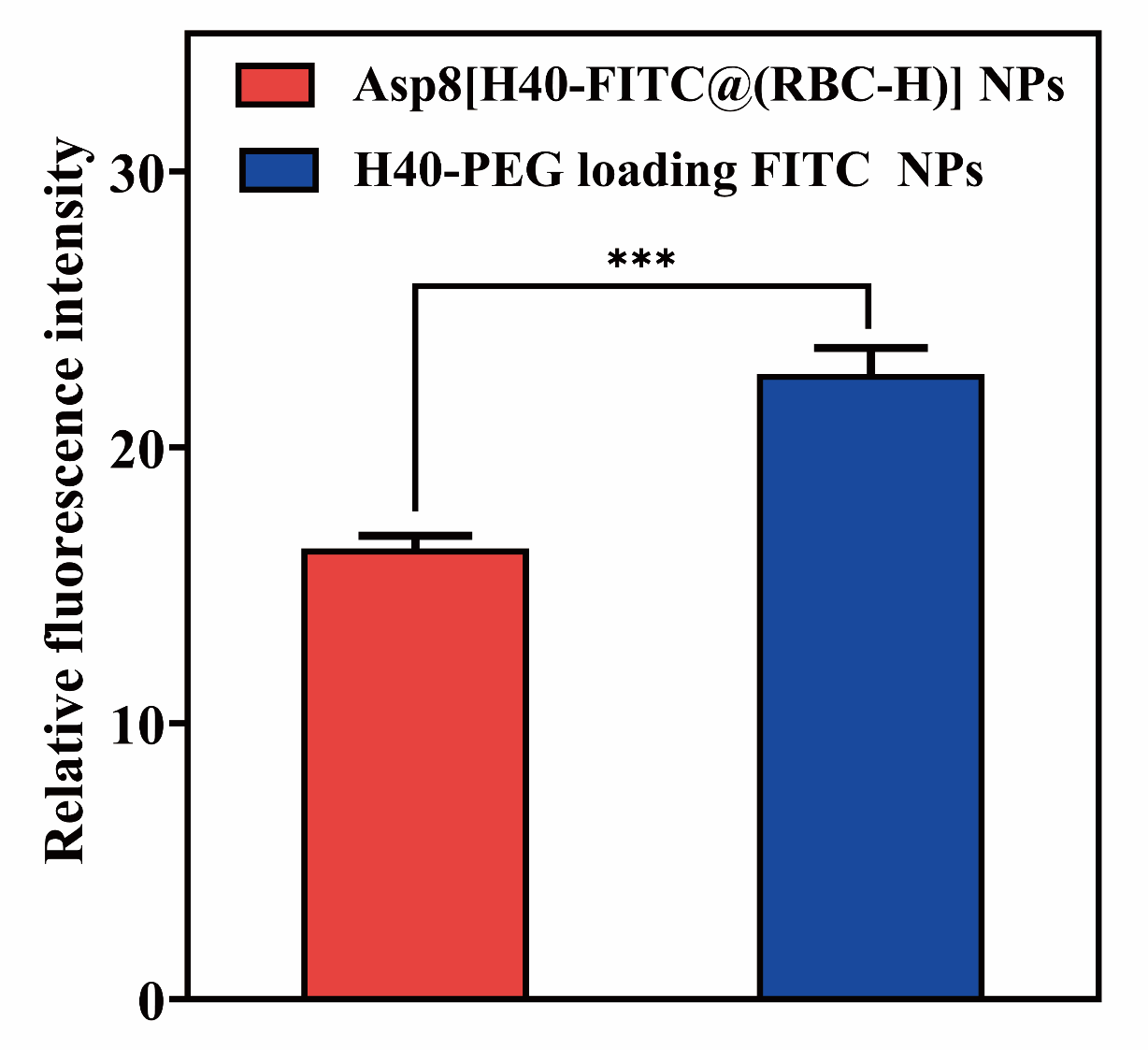


**Figure S2**. Quantitative analysis of the FITC fluorescence intensity. The data are presented the average ± standard deviation (n=3).


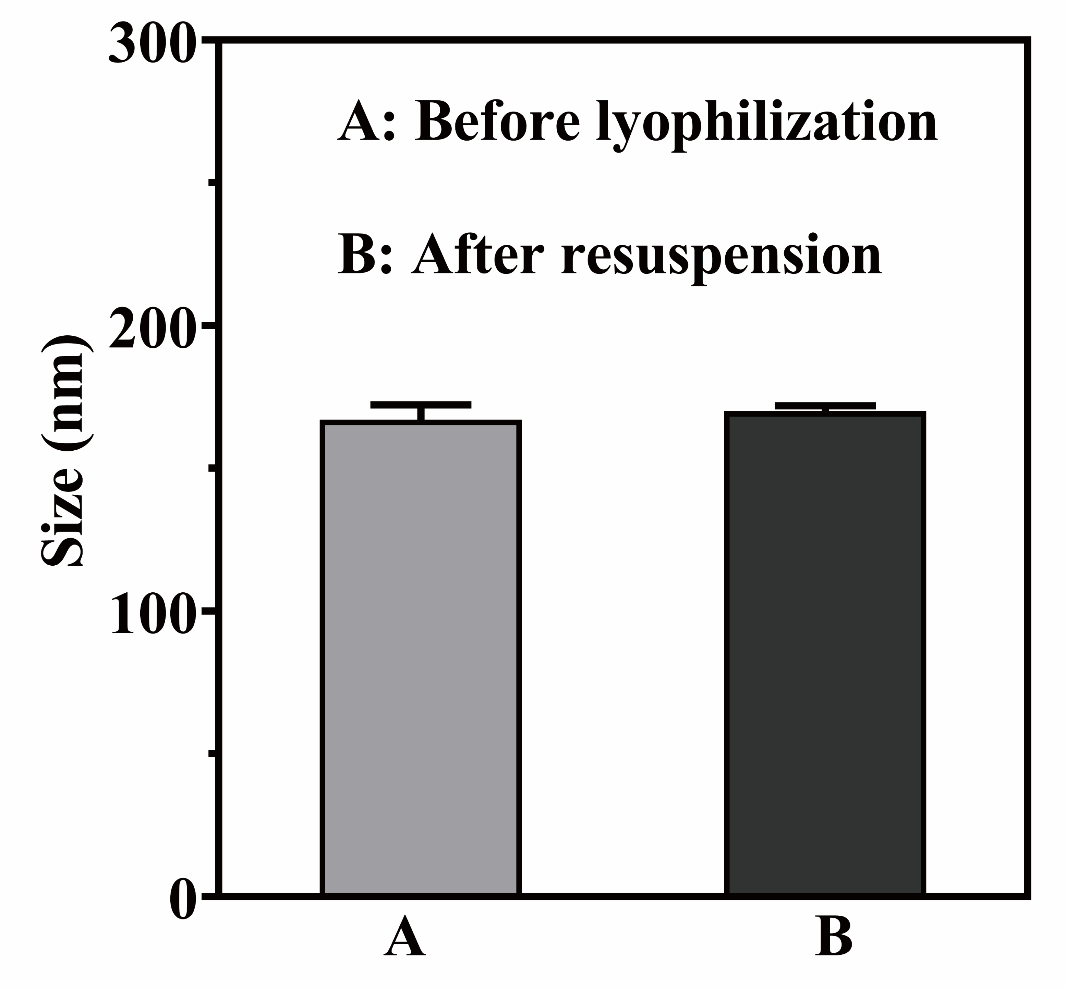


**Figure S3**. The average size of Asp8[H40-PEG@(RBC-H)] NPs was monitored by DLS before and after lyophilization. (mean ± SD; n=3).


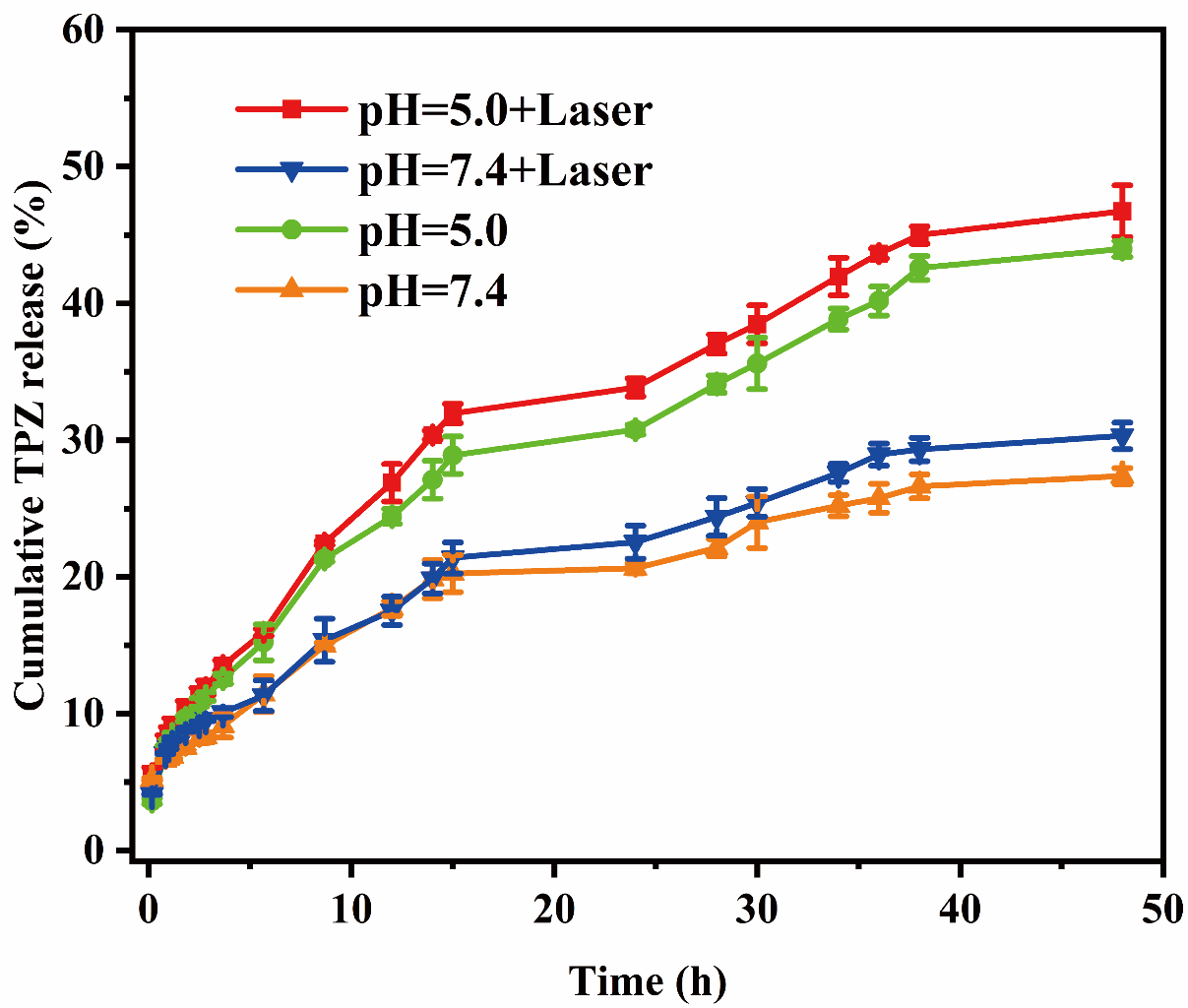


**Figure S4**. Release profiles of TPZ from the Asp8[H40-TPZ/IR780@(RBC-H)] NPs in different pH (5.0 and 7.4) buffer with or without 808 nm laser irradiation (1.0 W cm^-2^, 5 min). All data are exhibited as the average ± standard deviation (n=3).


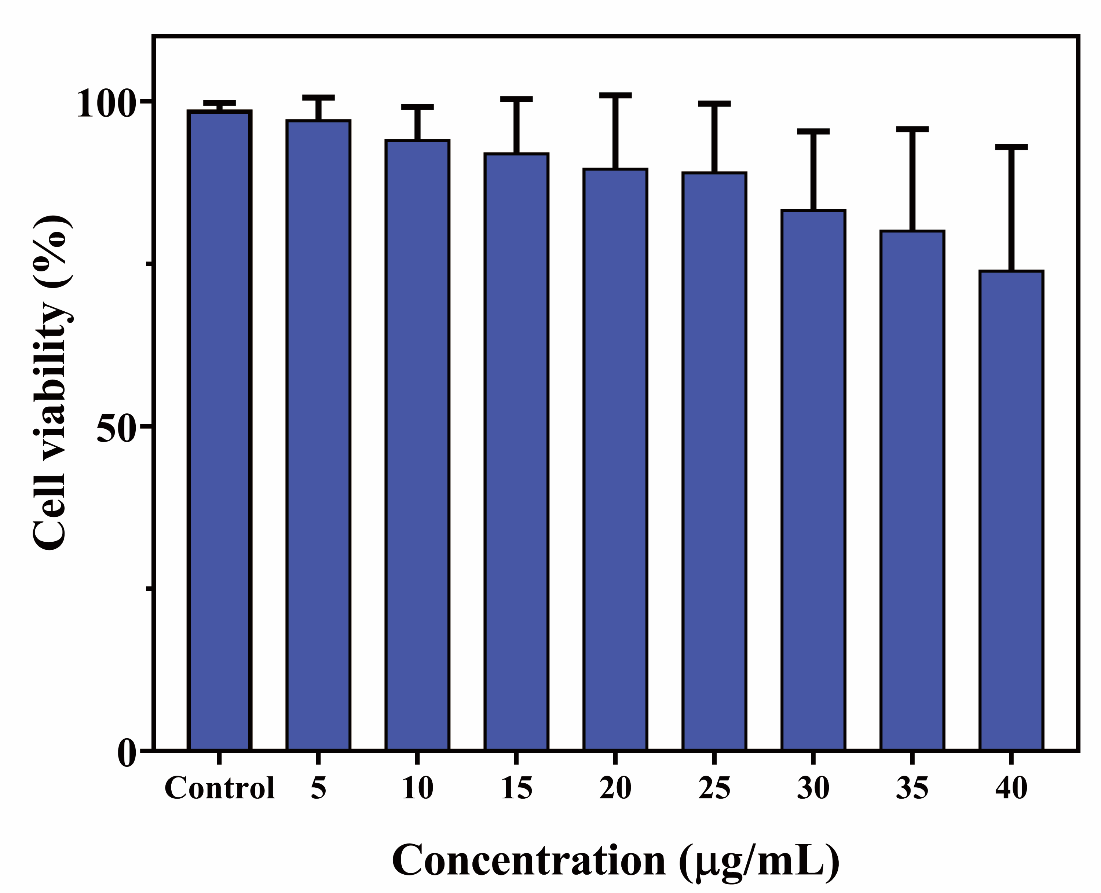


**Figure S5**. The cell viability of Asp8[H40-PEG@(RBC-H)] NPs against HUVEC cells after cultured for 72 h with series of concentrations. (mean ± SD; n=3).


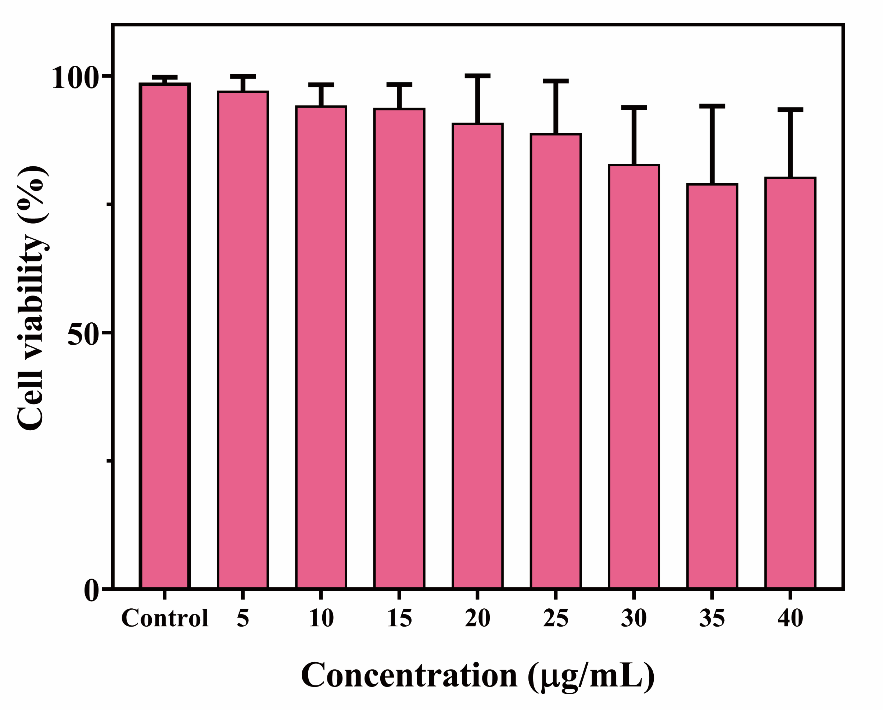


**Figure S6**. The cell viability of Asp8[H40-PEG@(RBC-H)] NPs against WSU-HN6 cells after cultured for 72 h with series of concentrations. (mean ± SD; n=3).


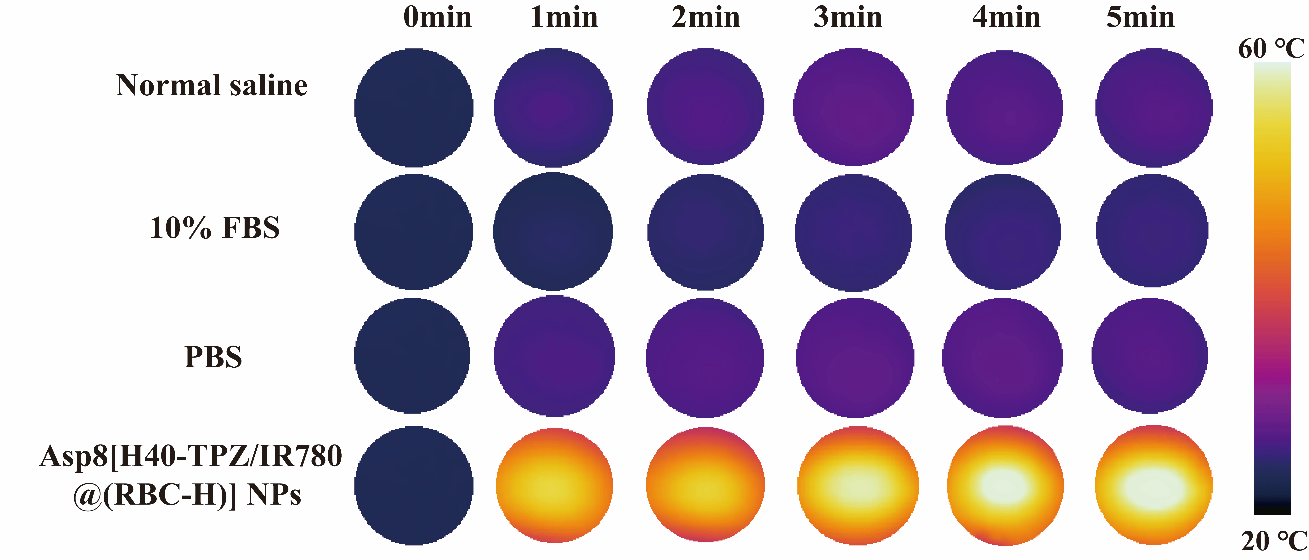


**Figure S7**. The increasing temperature photographs of normal saline, 10% FBS, PBS, and Asp8[H40-TPZ/IR780@(RBC-H)] NPs (the concentration of IR780 = 100 μg mL^-1^ ) upon 808 nm laser irradiation (1.0 W cm^-2^, 5min) *in vitro*.

**Supplementary References**

1. Chen H, Li G, Chi H, Wang D, Tu C, Pan L, et al. Alendronate-conjugated amphiphilic hyperbranched polymer based on Boltorn H40 and poly(ethylene glycol) for bone-targeted drug delivery. Bioconjug Chem. 2012;23:1915-24.

2. Li M, Fang H, Liu Q, Gai Y, Yuan L, Wang S, et al. Red blood cell membrane-coated upconversion nanoparticles for pretargeted multimodality imaging of triple-negative breast cancer. Biomater Sci. 2020;8:1802-14.

3. Wang H, Liu Y, He R, Xu D, Zang J, Weeranoppanant N, et al. Cell membrane biomimetic nanoparticles for inflammation and cancer targeting in drug delivery. Biomater Sci. 2020;8:552-68.

4. Gong C, Yu X, You B, Wu Y, Wang R, Han L, et al. Macrophage-cancer hybrid membrane-coated nanoparticles for targeting lung metastasis in breast cancer therapy. J Nanobiotechnology. 2020;18:92.
